# Supplementary material for: Pathogen infection and cholesterol deficiency activate the C. elegans p38 immune pathway through a TIR-1/SARM1 phase transition
Source: eLife. 2022 Jan 31;11:e74206. doi: 10.7554/eLife.74206 (PMC8923663; doi:10.7554/eLife.74206)
Supplement: Source data 1. [file elife-74206-data1.zip › Raw and annotated gel and blot images 2 of 2/Fig. 2 - figure supplement 1I and 1J.pdf]

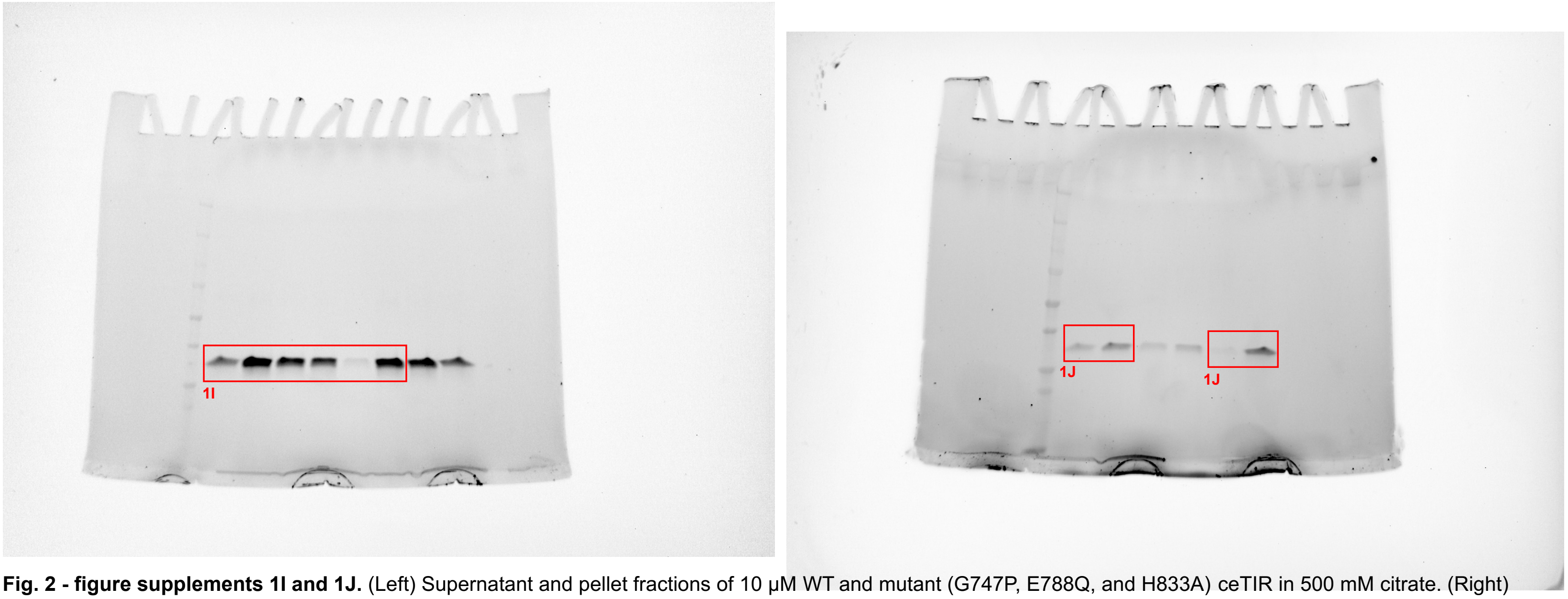

Fig. 2 - figure supplements 1I and 1J. (Left) Supernatant and pellet fractions of 10 µM WT and mutant (G747P, E788Q, and H833A) ceTIR in 500 mM citrate. (Right) Supernatant and pellet fractions of 3 µM WT and mutant (D773N and E788A) ceTIR in 500 mM citrate. For the images on the right, D773N was not included in the image for the body of the paper as this mutant was not of interest in this paper.
