# Supplementary material for: Pathogen infection and cholesterol deficiency activate the C. elegans p38 immune pathway through a TIR-1/SARM1 phase transition
Source: eLife. 2022 Jan 31;11:e74206. doi: 10.7554/eLife.74206 (PMC8923663; doi:10.7554/eLife.74206)
Supplement: Source data 1. [file elife-74206-data1.zip › Raw and annotated gel and blot images 2 of 2/Fig. 2 - figure supplement 1G_Annotated.pdf]

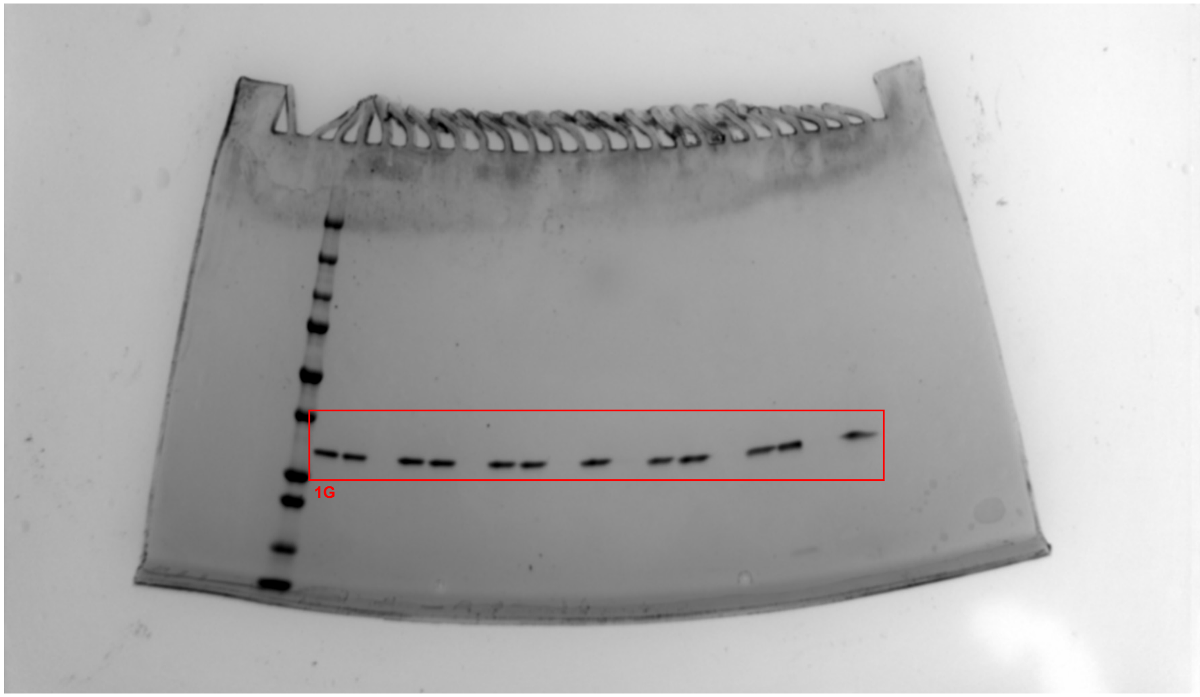

Figure 2 - figure supplement 1G. Precentrifugation control, supernatant, and pelleted fractions of 5 μM ceTIR in 0, 125, 250, 500, 750, and 1000 mM citrate.
