# Supplementary material for: Pathogen infection and cholesterol deficiency activate the C. elegans p38 immune pathway through a TIR-1/SARM1 phase transition
Source: eLife. 2022 Jan 31;11:e74206. doi: 10.7554/eLife.74206 (PMC8923663; doi:10.7554/eLife.74206)
Supplement: Source data 1. [file elife-74206-data1.zip › Raw and annotated gel and blot images 2 of 2/Fig. 2 - figure supplement 2C_Annotated.pdf]

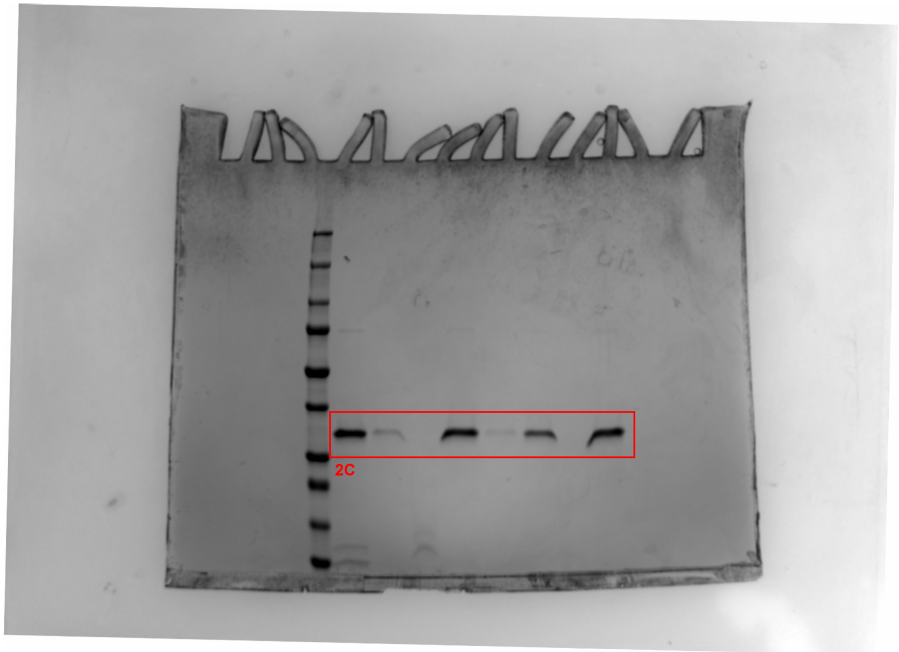

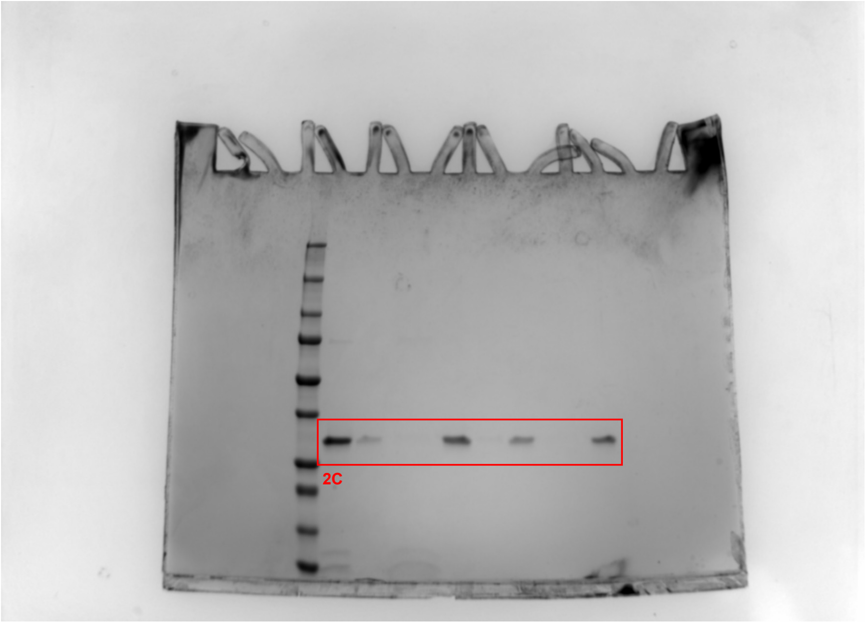

Fig. 2 - figure supplement 2C. (Left) Supernatant and pellet fractions of 5 µM TIR without and with 25% PEG 3350 after one round of centrifugation, and without and with 25% PEG 3350 after centrifugation of the resuspension. (Right) Supernatant and pellet fractions of 5 µM TIR without and with 500 mM citrate after one round of centrifugation, and without and with 500 mM citrate after centrifugation of the resuspension.
