# Supplementary material for: Pathogen infection and cholesterol deficiency activate the C. elegans p38 immune pathway through a TIR-1/SARM1 phase transition
Source: eLife. 2022 Jan 31;11:e74206. doi: 10.7554/eLife.74206 (PMC8923663; doi:10.7554/eLife.74206)
Supplement: Source data 1. [file elife-74206-data1.zip › Raw and annotated gel and blot images 2 of 2/Fig. 2 - figure supplement 2A and 2B.pdf]

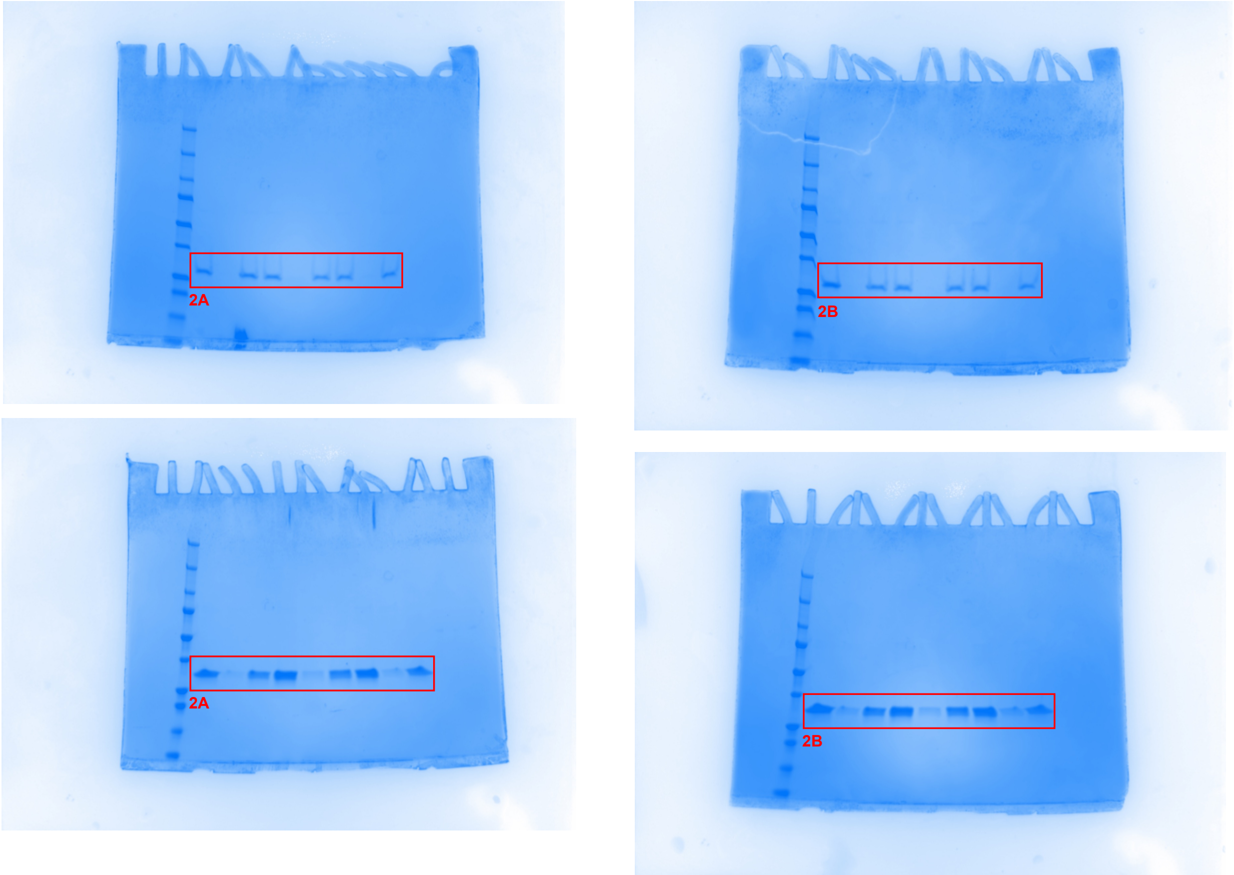

Fig. 2 - figure supplements 2A and 2B. (Left top) Precentrifugation control, supernatant, and pelleted fractions of 10 μM ceTIR n 25% PEG 3350, where 0, 1, or 2% 1,6-hexanediol was added after precipitate formation. (Left bottom) Precentrifugation control, supernatant, and pelleted fractions of 10 μM ceTIR in 500 mM citrate, where 0, 1, or 2% 1,-6 hexanediol was added after precipitate formation.(Right top) Precentrifugation control, supernatant, and pelleted fractions of 10 μM ceTIR in 25% PEG 3350, where 0, 1, or 2% 1,6-hexanediol was added before precipitate formation. (Right bottom) Precentrifugation control, supernatant, and pelleted fractions of 10 μM ceTIR in 500 mM citrate, where 0, 1, or 2% 1,6-hexanediol was added before precipitate formation.
