# Supplementary material for: Pathogen infection and cholesterol deficiency activate the C. elegans p38 immune pathway through a TIR-1/SARM1 phase transition
Source: eLife. 2022 Jan 31;11:e74206. doi: 10.7554/eLife.74206 (PMC8923663; doi:10.7554/eLife.74206)
Supplement: Source data 1. [file elife-74206-data1.zip › Raw and annotated gel and blot images 2 of 2/Fig. 2 - figure supplement 2D_Annotated.pdf]

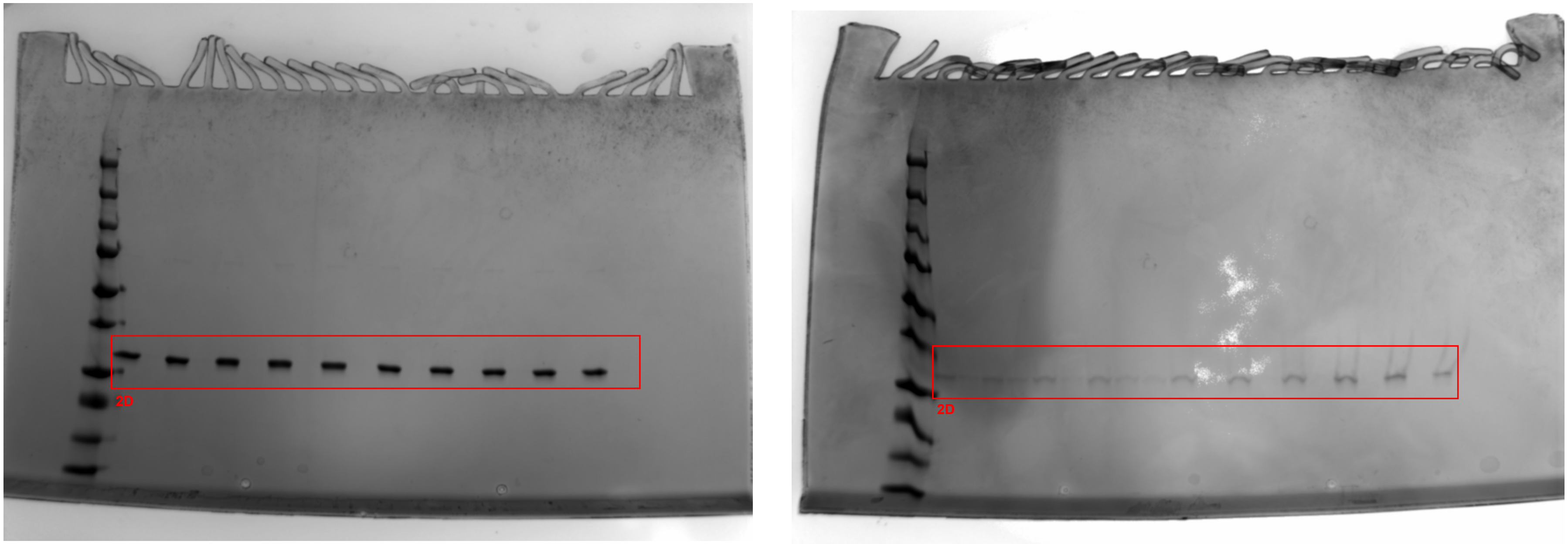

Fig. 2 - figure supplement 2D (Left) Supernatant and pellet fractions of 5 μM ceTIR in the absence of 25% PEG 3350, where pH increases from 4.5-9. (Right) Supernatant and pellet fraction of 5 μM ceTIR in the presence of 25% PEG 3350, where pH increases from 4.5-9.
