# Supplementary figures and images for: Pathogen infection and cholesterol deficiency activate the C. elegans p38 immune pathway through a TIR-1/SARM1 phase transition
Source: eLife. 2022 Jan 31;11:e74206. doi: 10.7554/eLife.74206 (PMC8923663; doi:10.7554/eLife.74206)

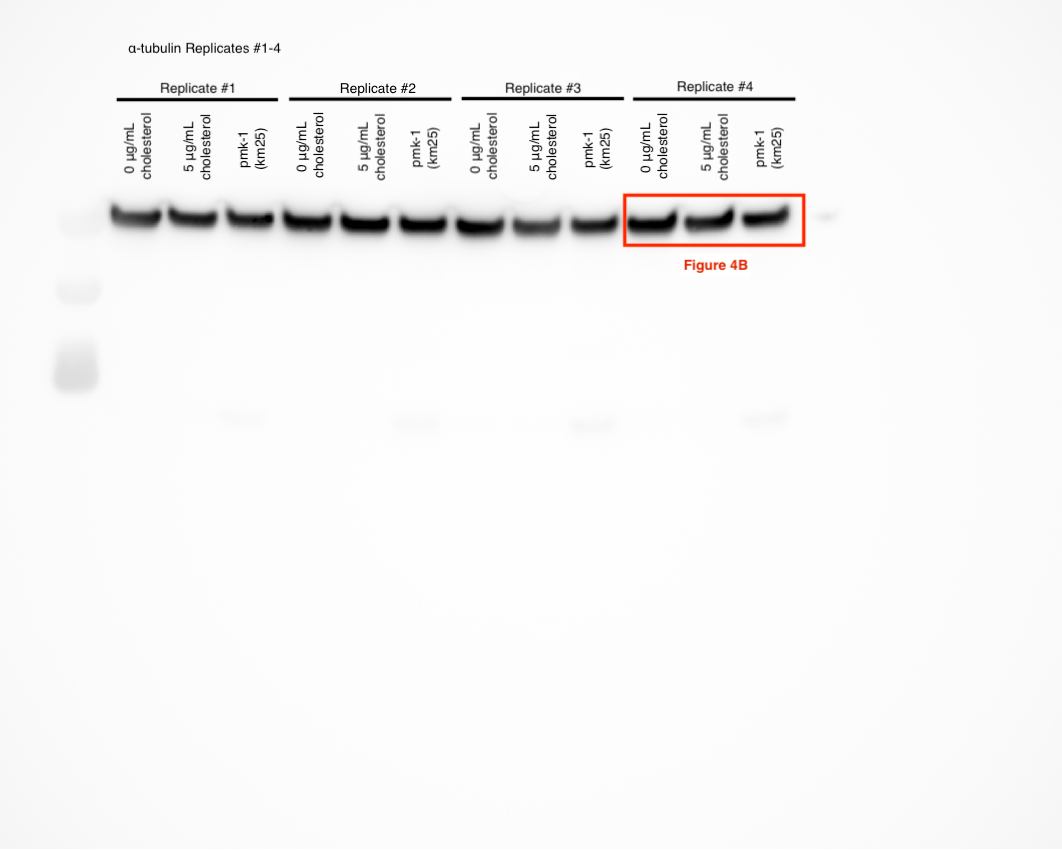

Supplement: Source data 1. [file elife-74206-data1.zip › Raw and annotated gel and blot images 2 of 2/Fig. 4B alpha_tubulin_Annotated_Replicates#1-4.tif]

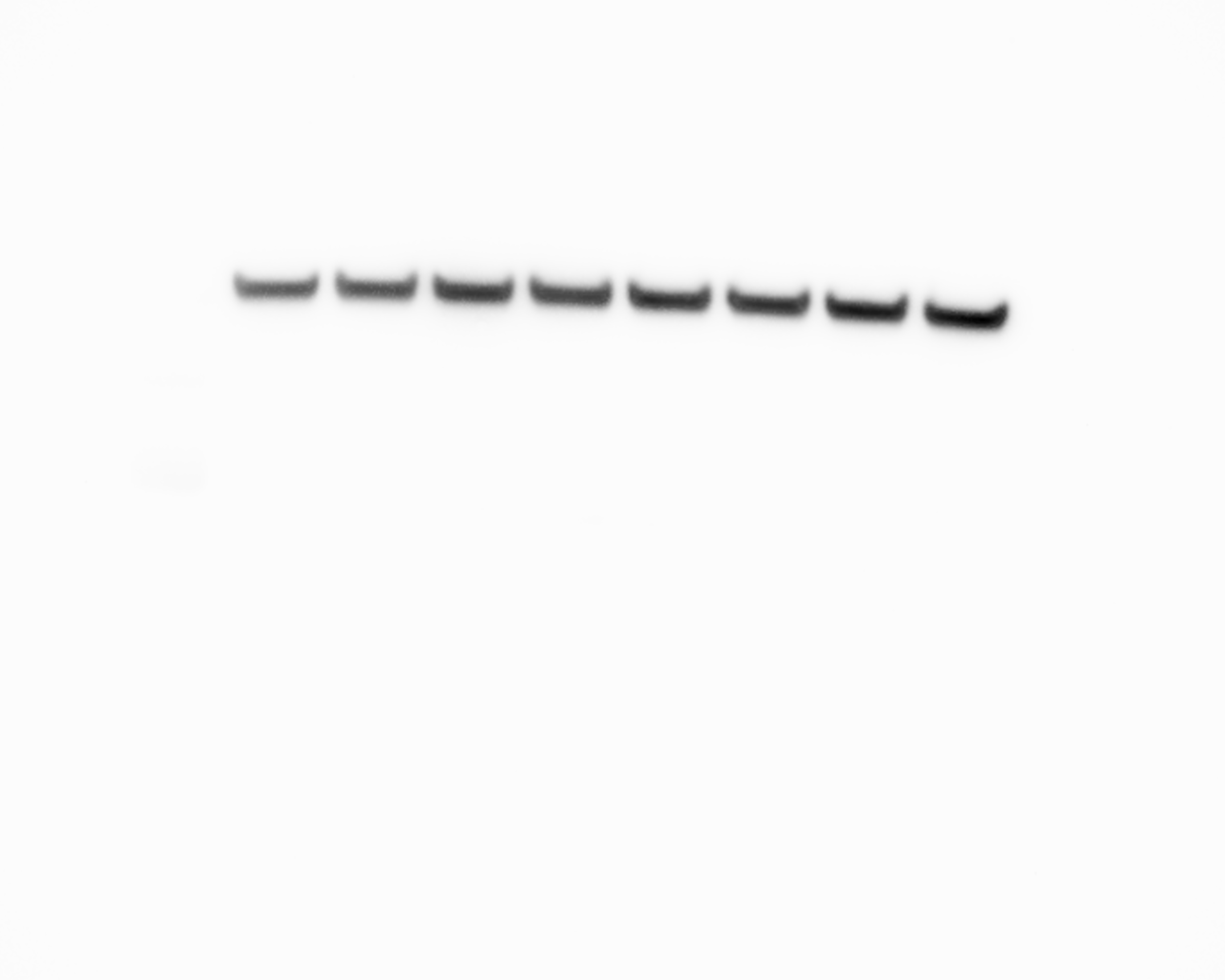

Supplement: Source data 1. [file elife-74206-data1.zip › Raw and annotated gel and blot images 2 of 2/Fig. 4E Alpha tubulin_raw_Replicates#1-2.tif]

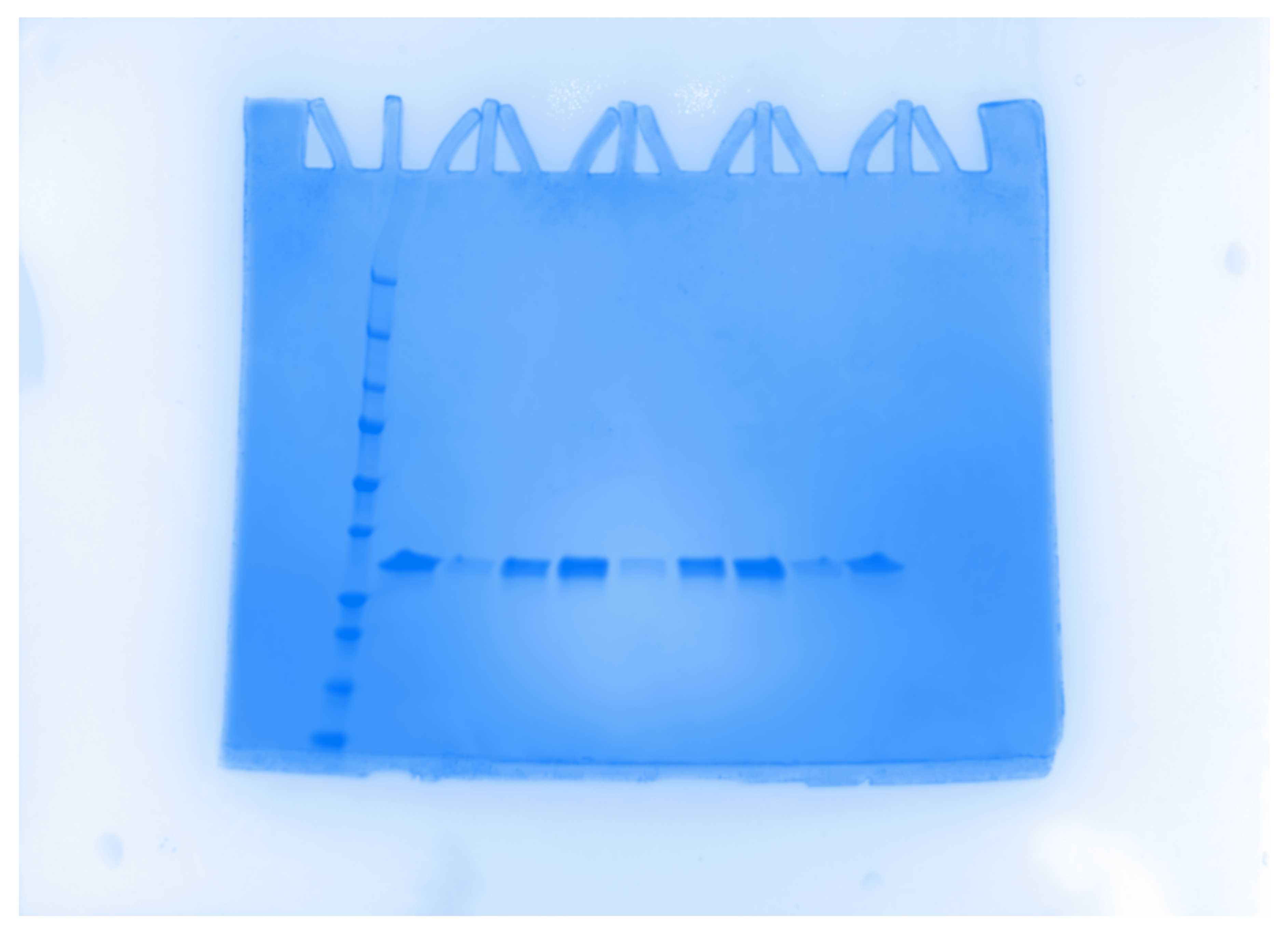

Supplement: Source data 1. [file elife-74206-data1.zip › Raw and annotated gel and blot images 2 of 2/Fig. 2 - figure supplement 2B_Citrate_raw.png]

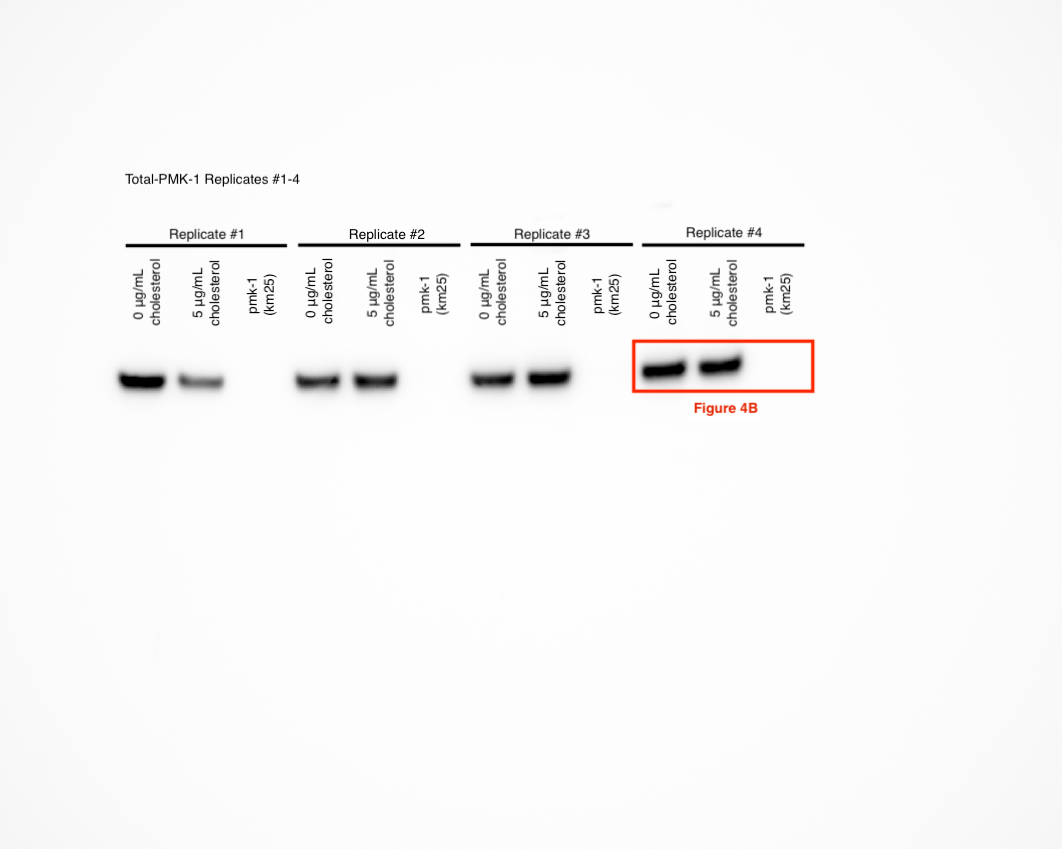

Supplement: Source data 1. [file elife-74206-data1.zip › Raw and annotated gel and blot images 2 of 2/Fig. 4B Total-p38_Annotated_Replicates#1-4.tif]

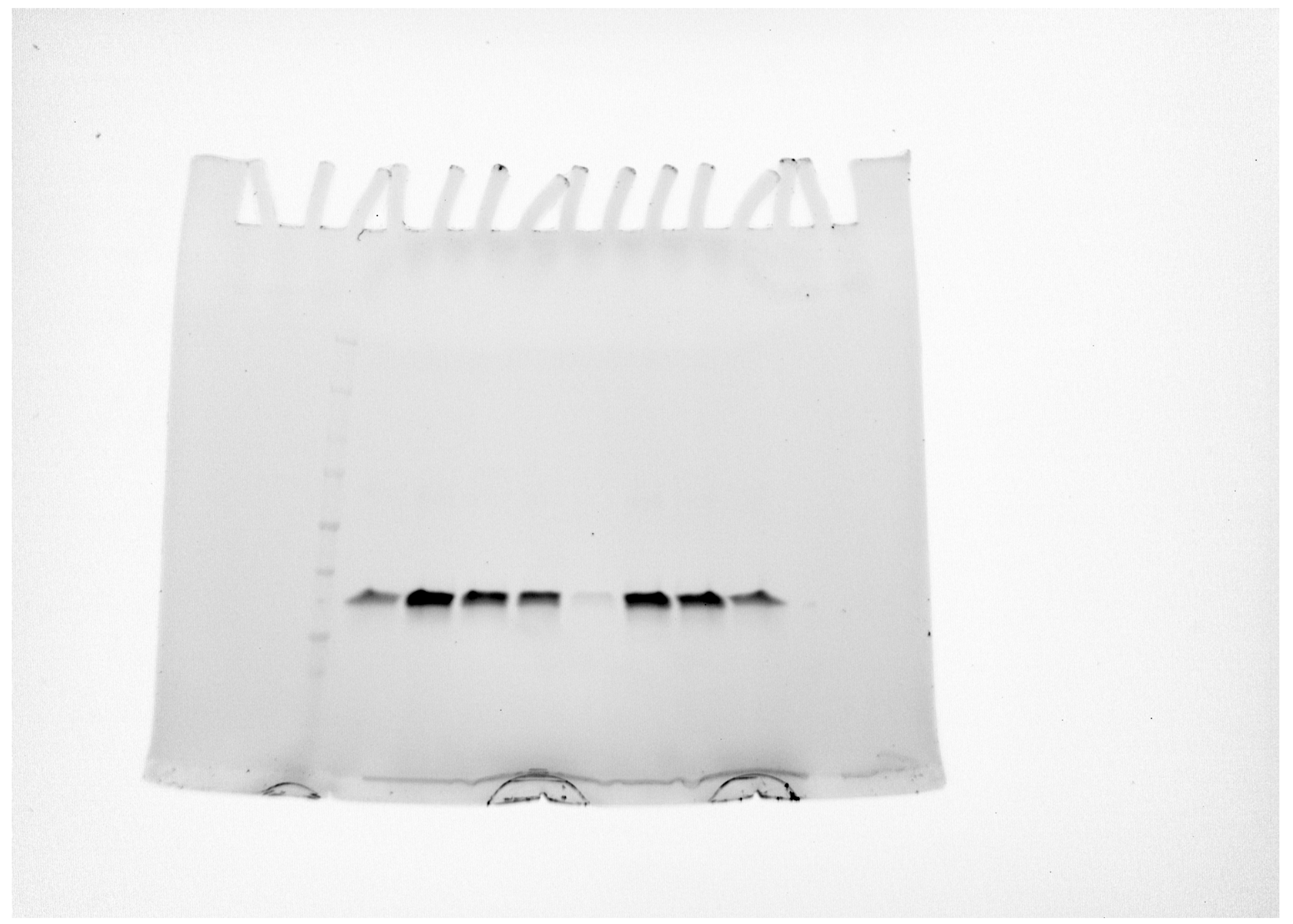

Supplement: Source data 1. [file elife-74206-data1.zip › Raw and annotated gel and blot images 2 of 2/Fig. 2 - figure supplement 1I_raw.png]

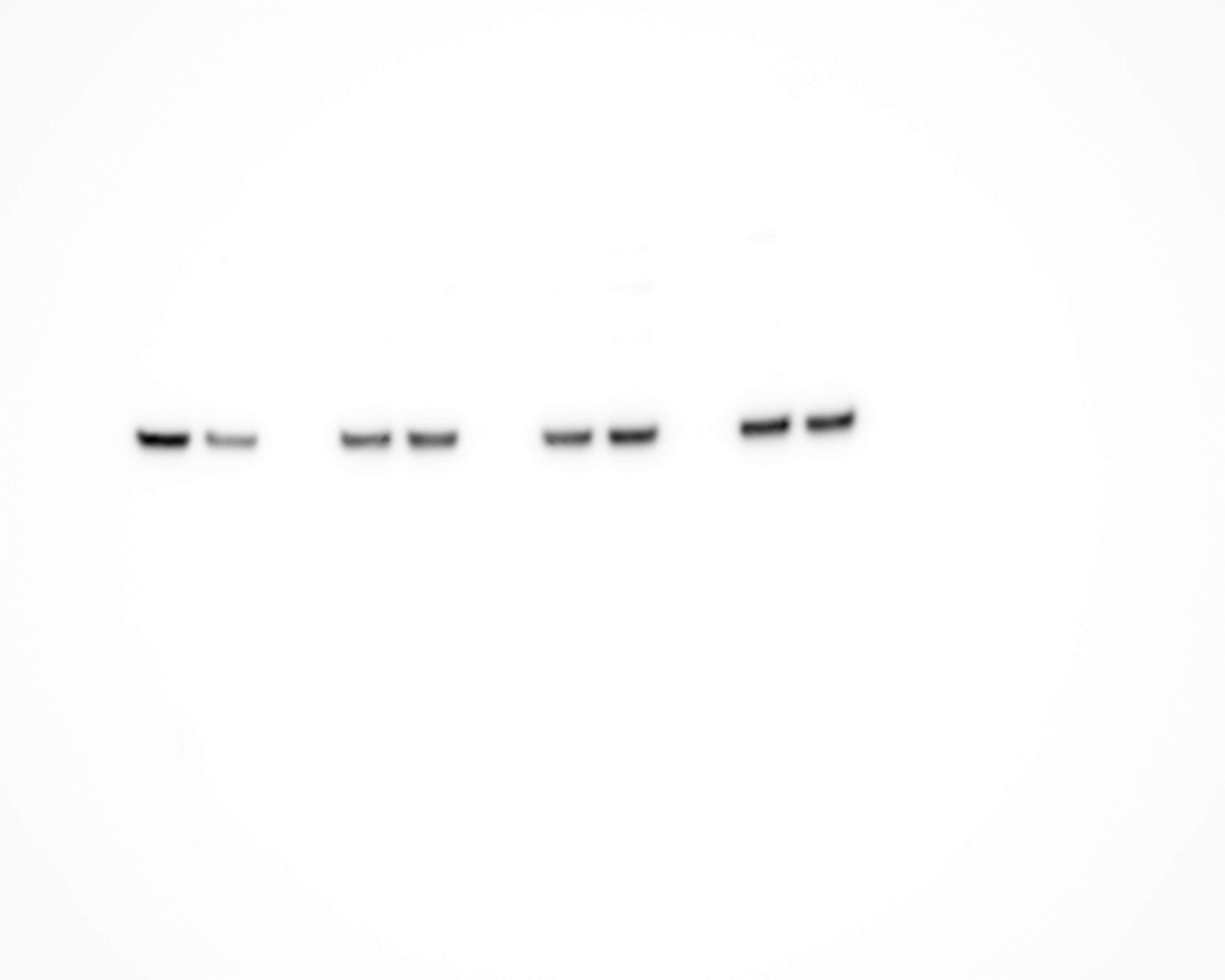

Supplement: Source data 1. [file elife-74206-data1.zip › Raw and annotated gel and blot images 2 of 2/Fig. 4B Total-p38_raw_Replicates#1-4.tif]

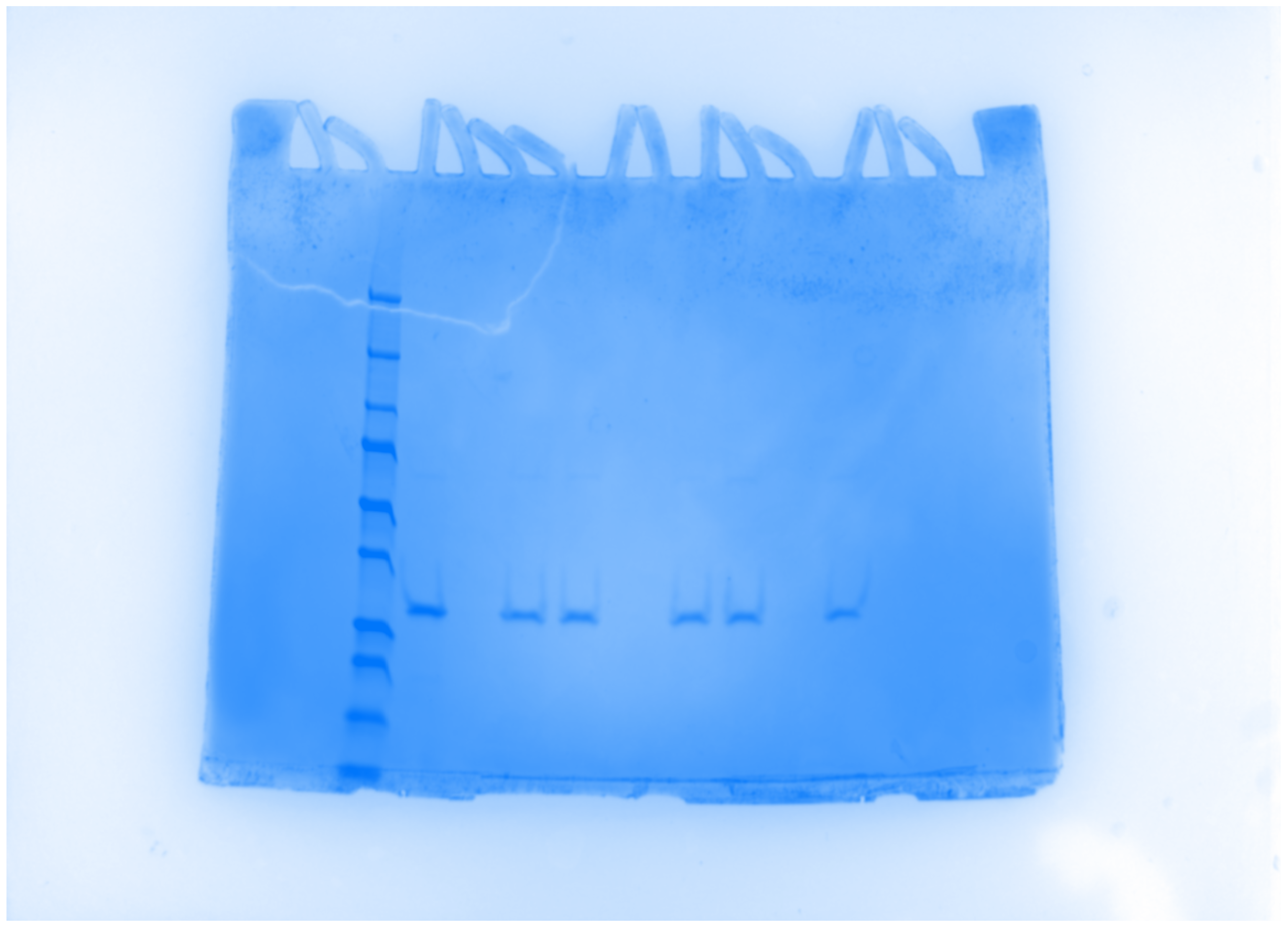

Supplement: Source data 1. [file elife-74206-data1.zip › Raw and annotated gel and blot images 2 of 2/Fig. 2 - figure supplement 2B_PEG_raw.png]

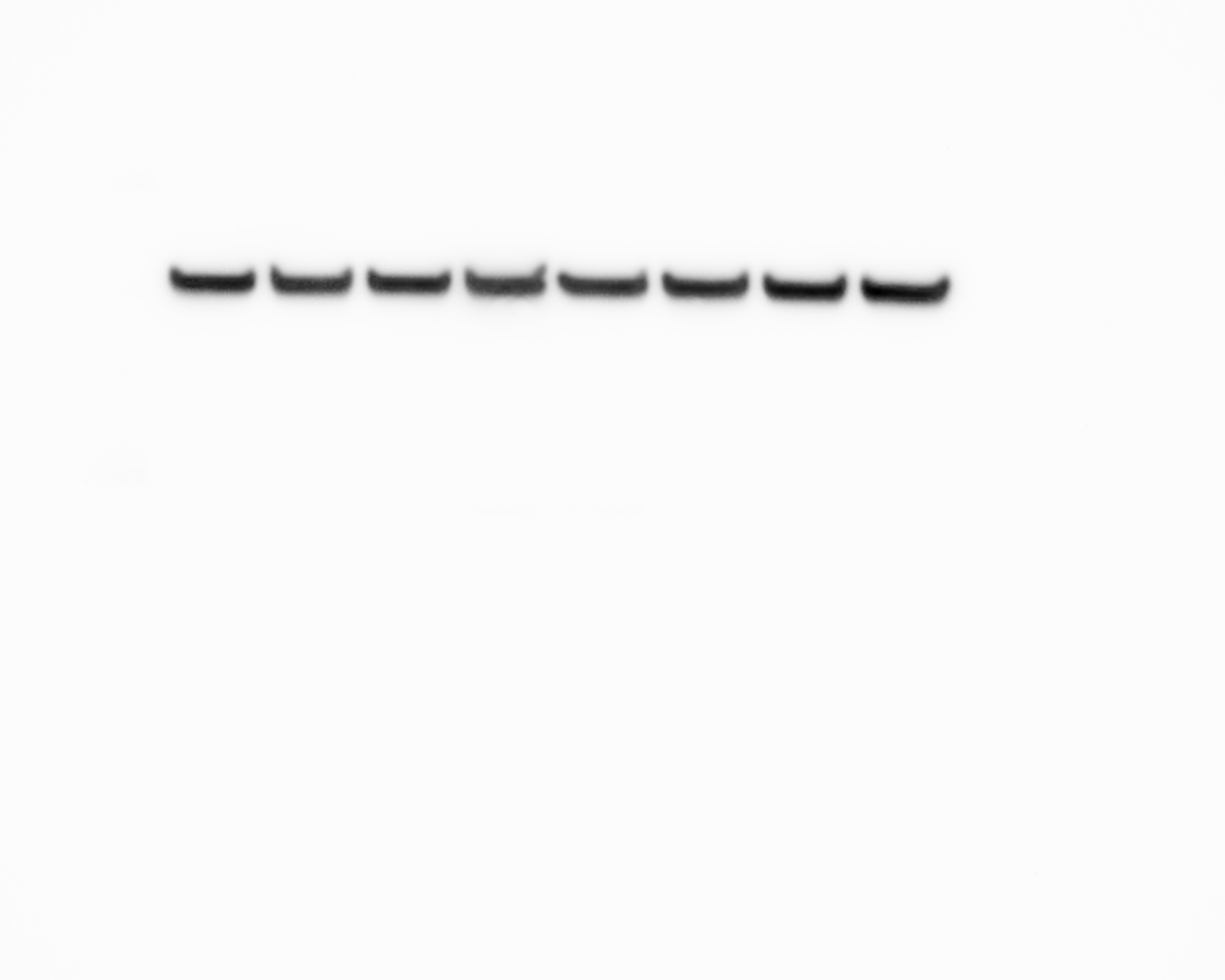

Supplement: Source data 1. [file elife-74206-data1.zip › Raw and annotated gel and blot images 2 of 2/Fig. 4E Alpha tubulin_raw_Replicates#3-4.tif]

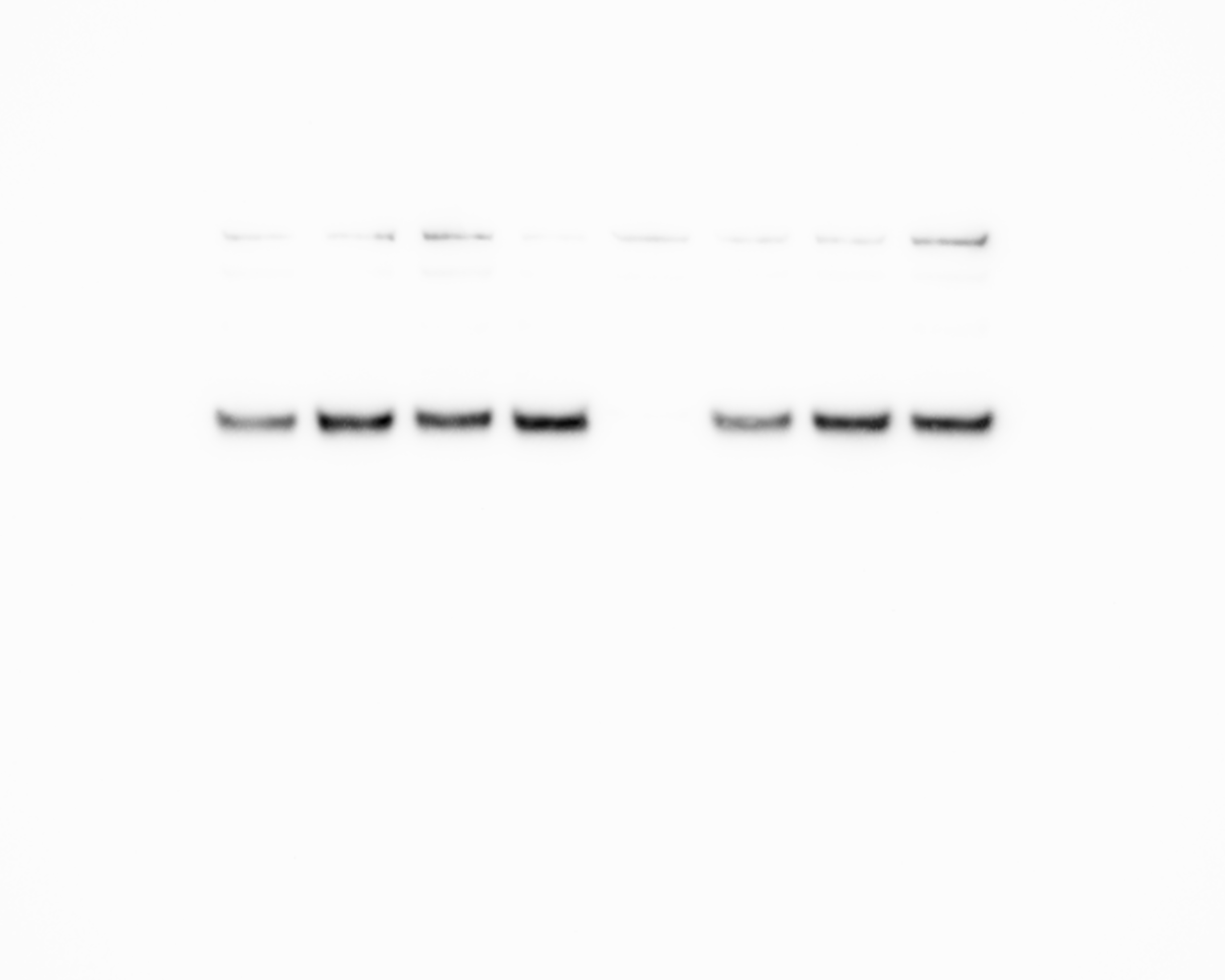

Supplement: Source data 1. [file elife-74206-data1.zip › Raw and annotated gel and blot images 2 of 2/Fig. 4E Total-p38_raw_Replicates#3-4.tif]

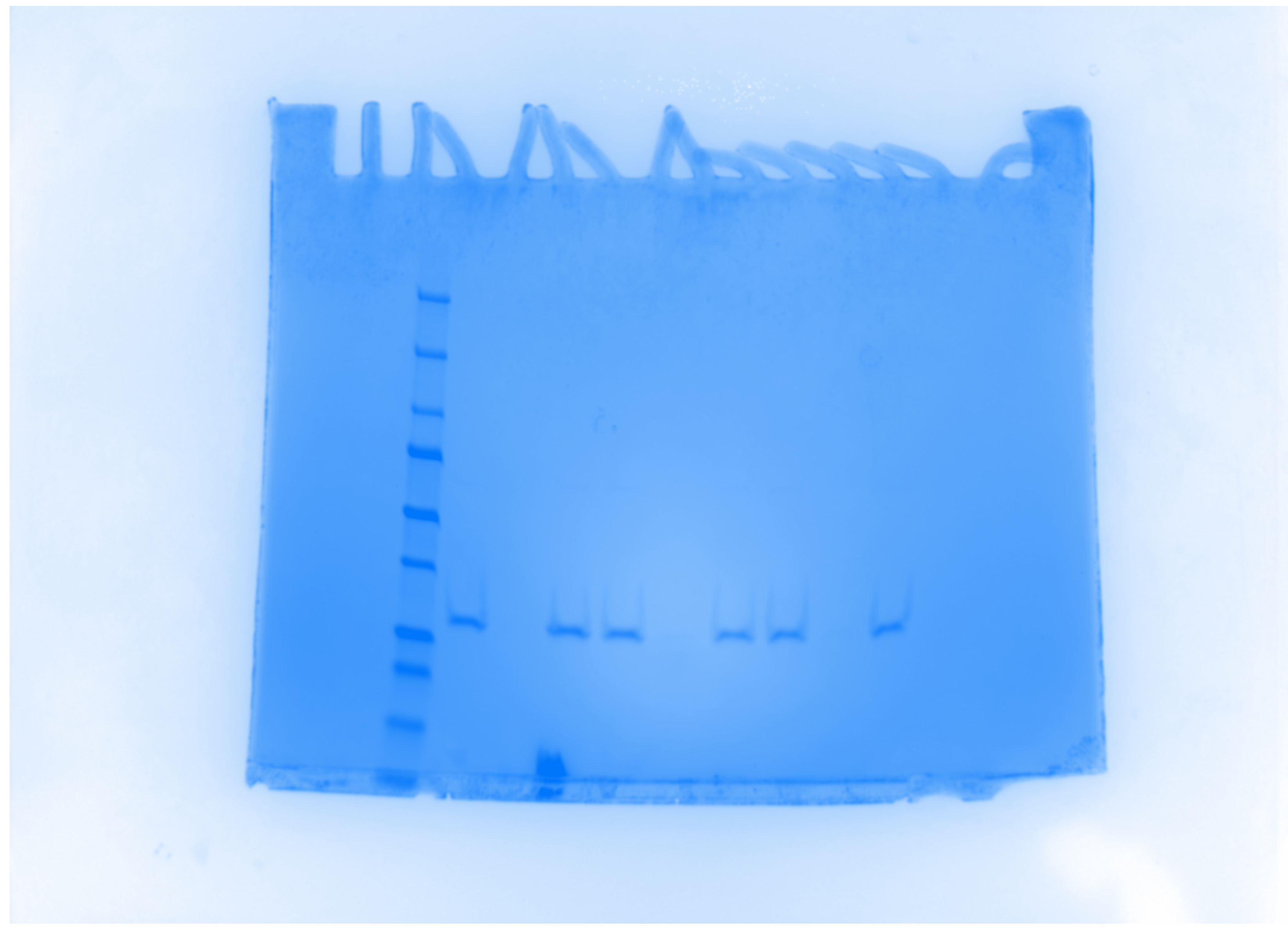

Supplement: Source data 1. [file elife-74206-data1.zip › Raw and annotated gel and blot images 2 of 2/Fig. 2 - figure supplement 2A_PEG_raw.png]

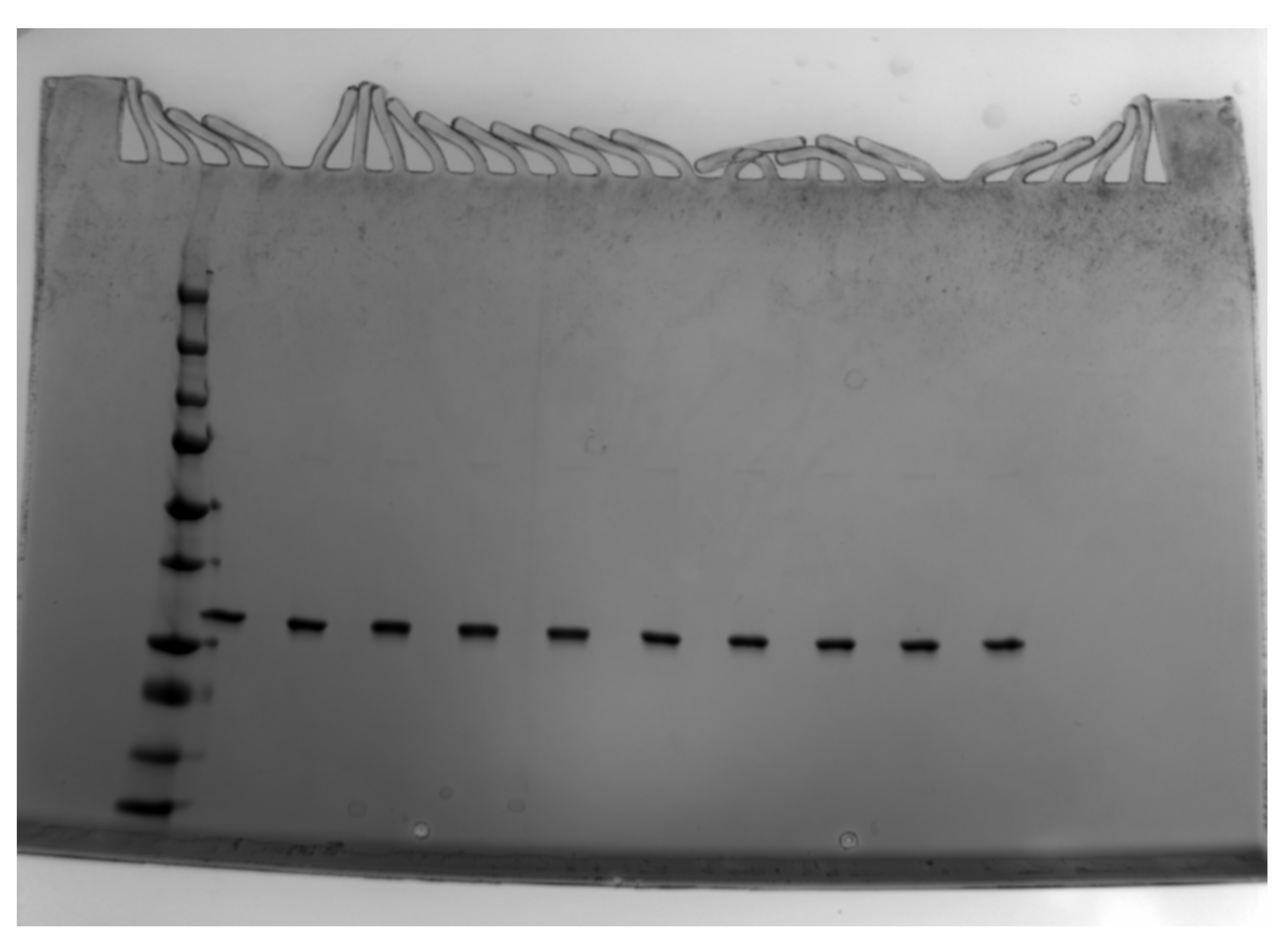

Supplement: Source data 1. [file elife-74206-data1.zip › Raw and annotated gel and blot images 2 of 2/Fig. 2 - figure supplement 2D_NoAddition_raw.png]

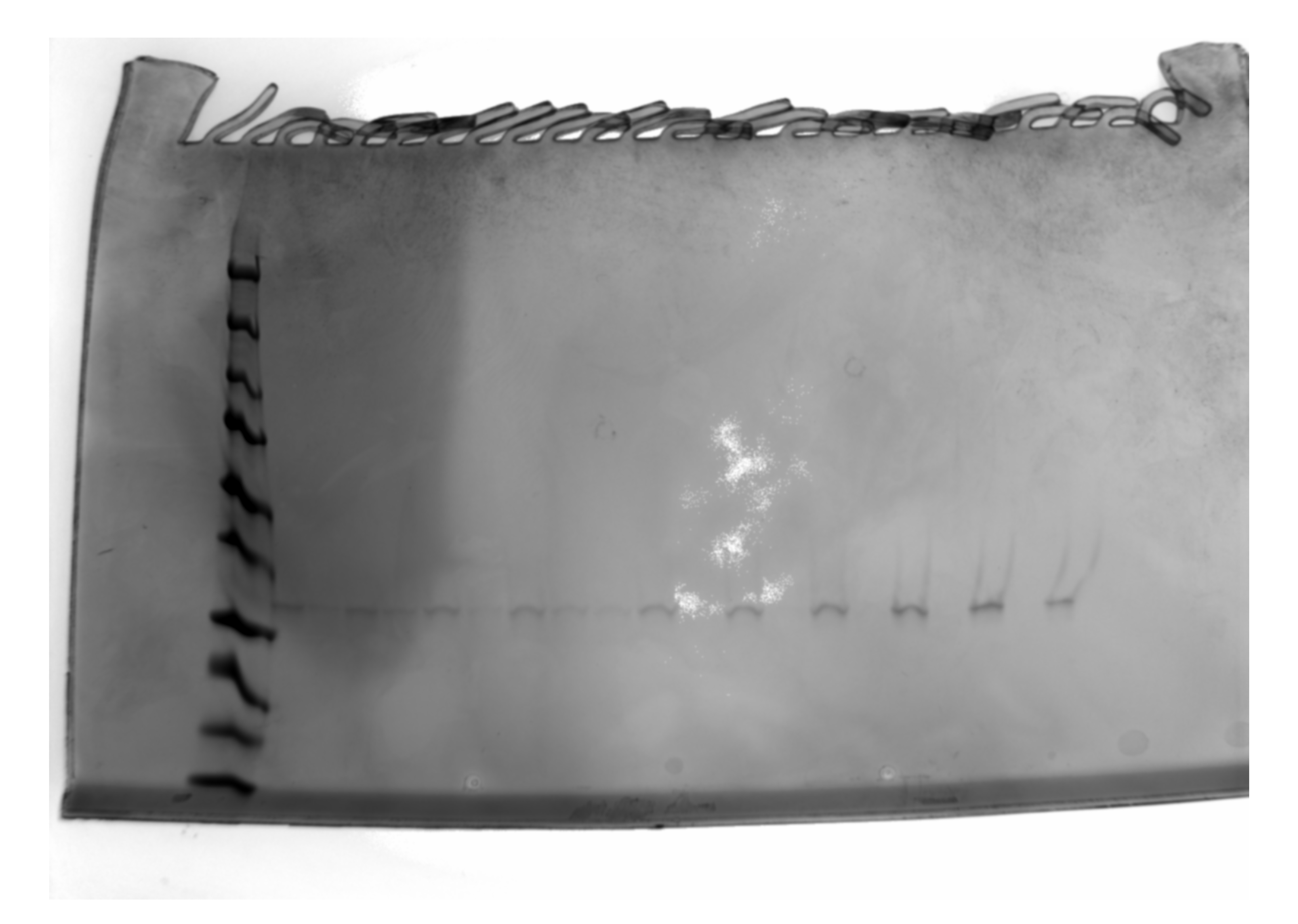

Supplement: Source data 1. [file elife-74206-data1.zip › Raw and annotated gel and blot images 2 of 2/Fig. 2 - figure supplement 2D_PEG_raw.png]

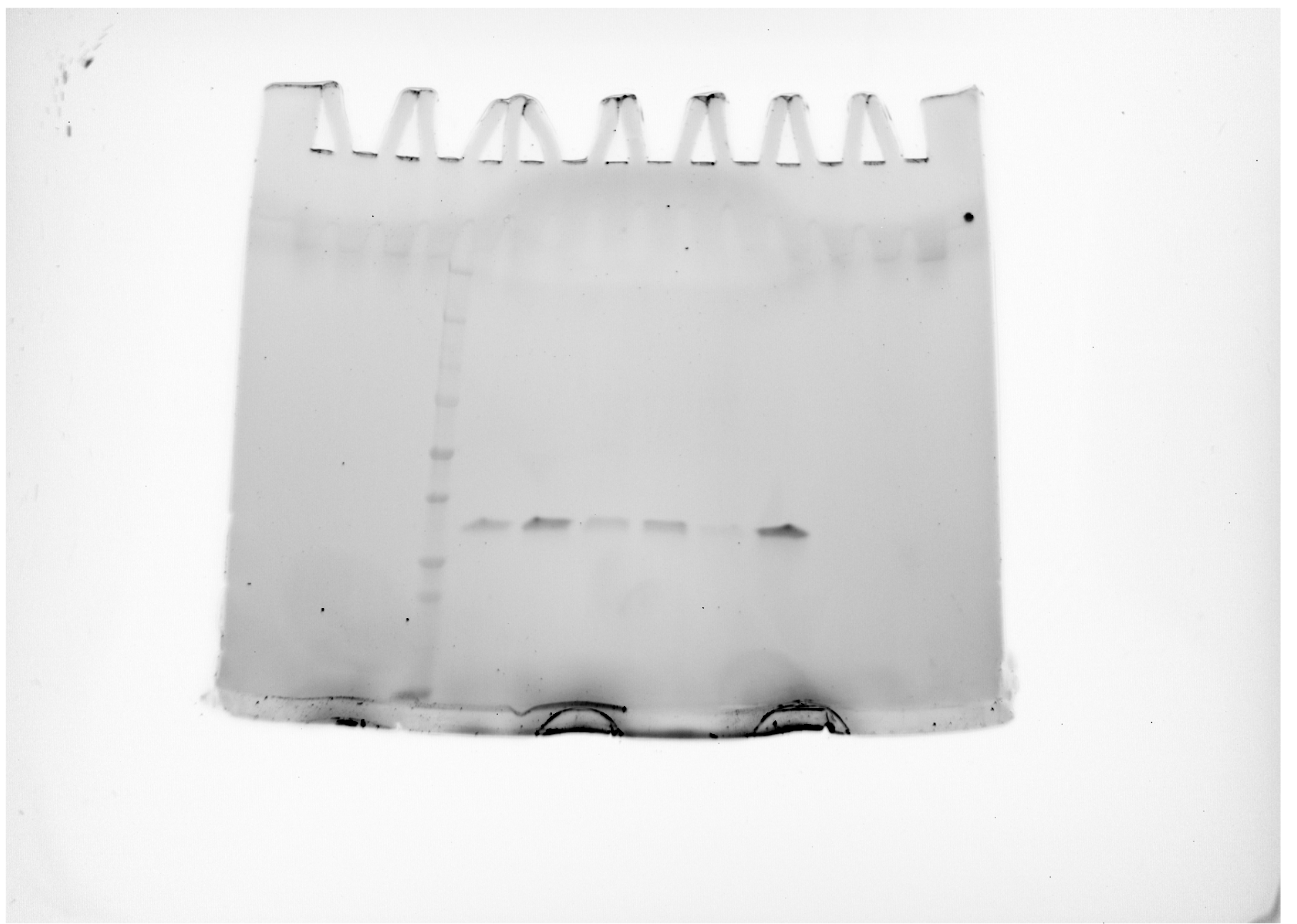

Supplement: Source data 1. [file elife-74206-data1.zip › Raw and annotated gel and blot images 2 of 2/Fig. 2 - figure supplement 1J_raw.png]

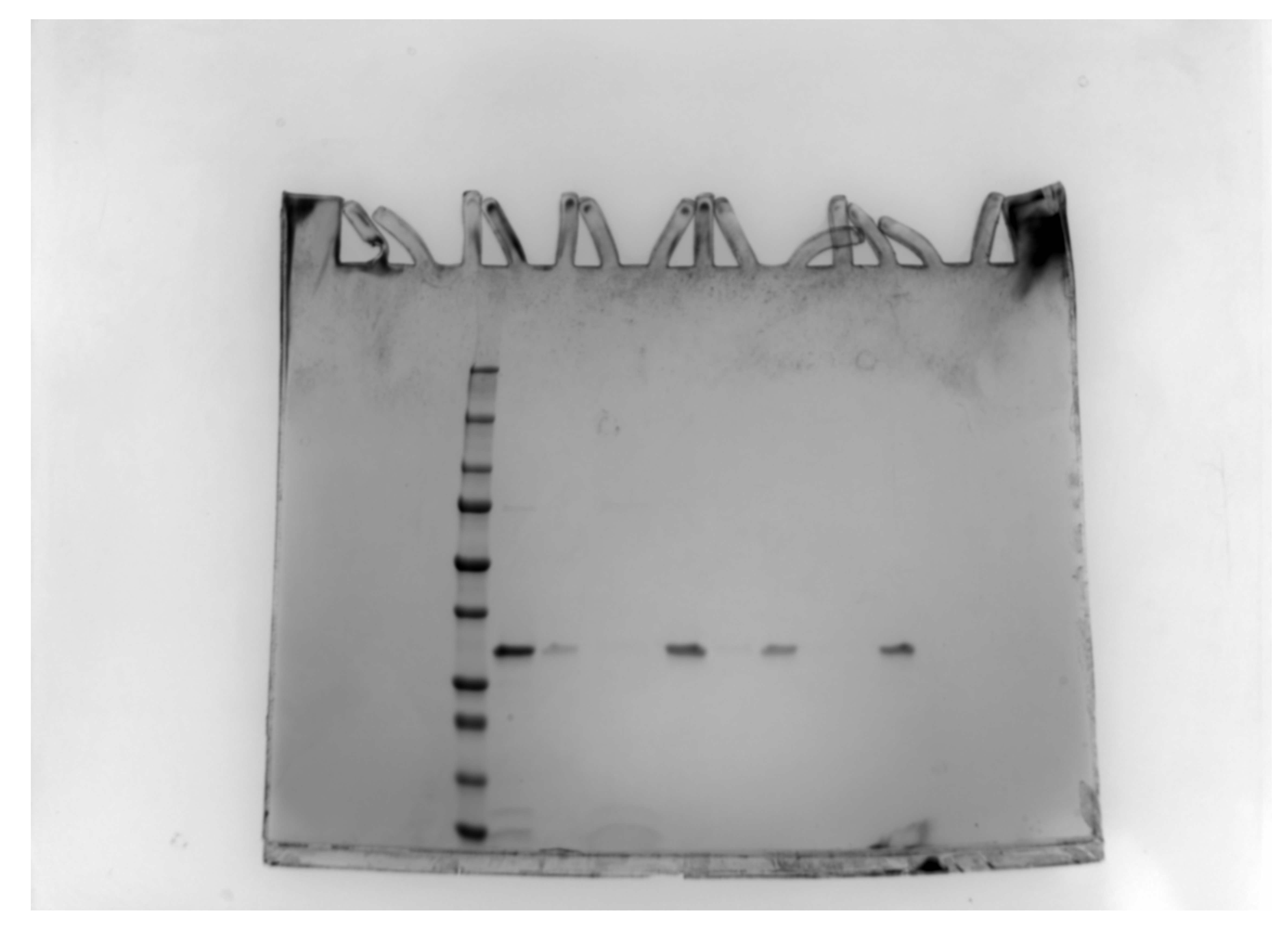

Supplement: Source data 1. [file elife-74206-data1.zip › Raw and annotated gel and blot images 2 of 2/Fig. 2 - figure supplement 2C_Citrate_raw.png]

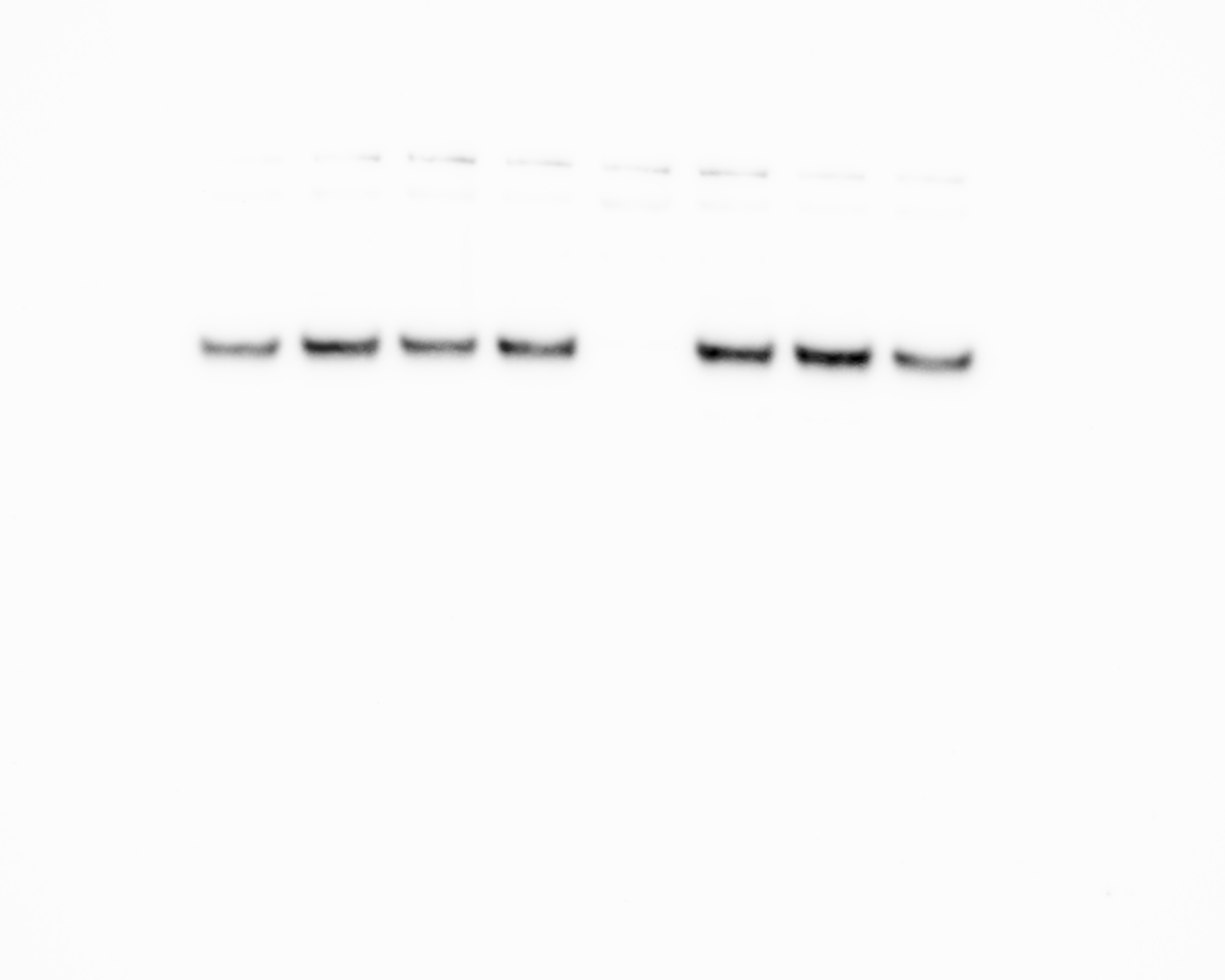

Supplement: Source data 1. [file elife-74206-data1.zip › Raw and annotated gel and blot images 2 of 2/Fig. 4E Total-p38_raw_Replicates#1-2.tif]

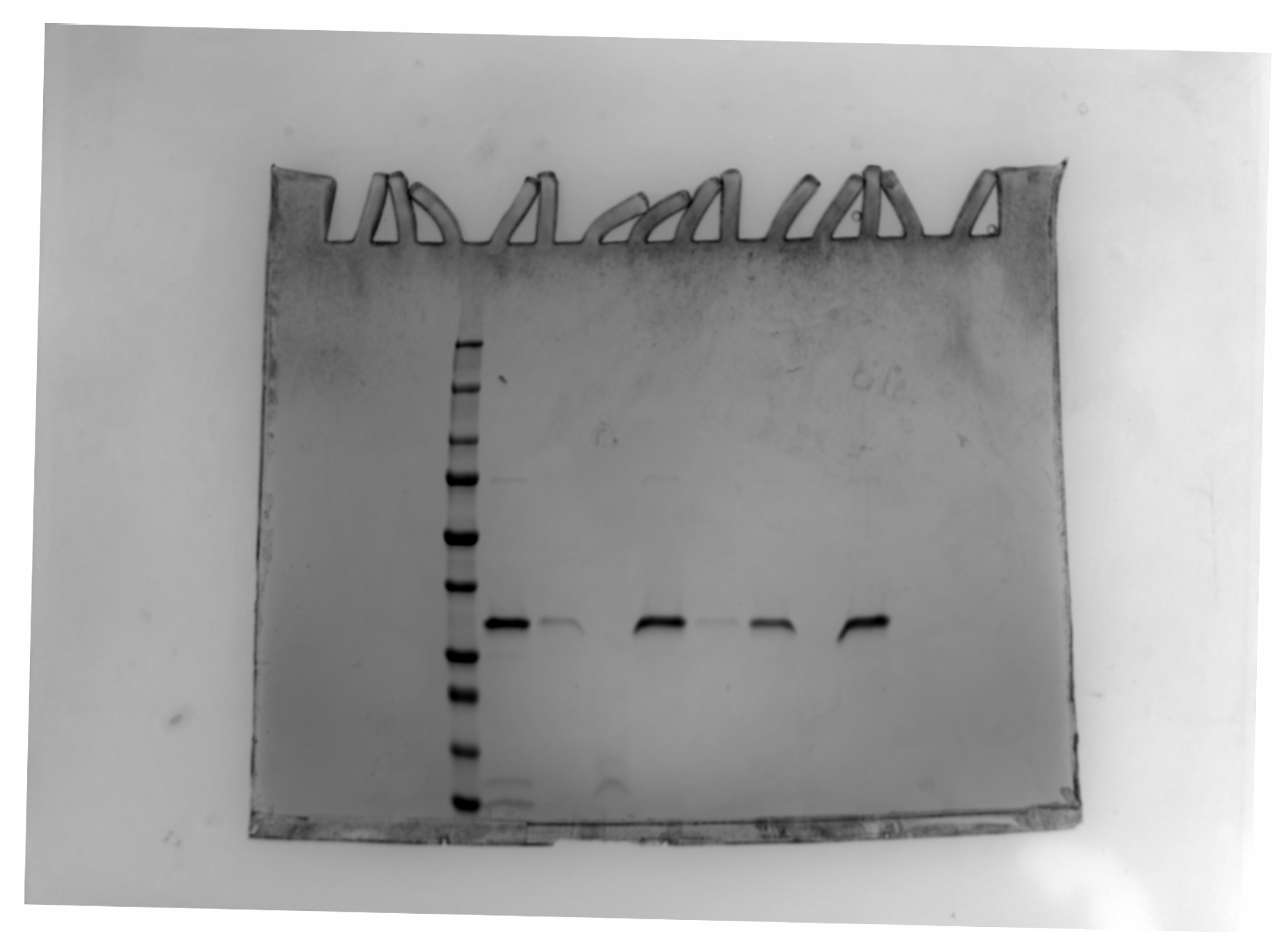

Supplement: Source data 1. [file elife-74206-data1.zip › Raw and annotated gel and blot images 2 of 2/Fig. 2 - figure supplement 2C_PEG_raw.png]

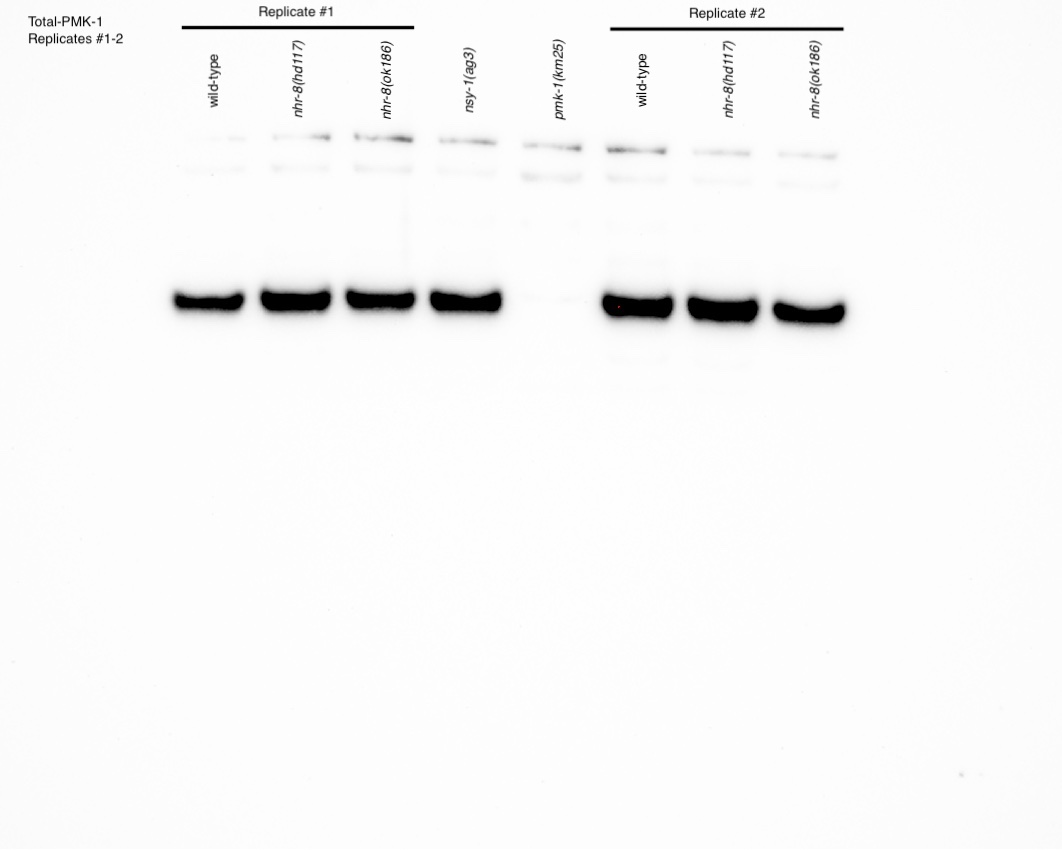

Supplement: Source data 1. [file elife-74206-data1.zip › Raw and annotated gel and blot images 2 of 2/Fig. 4E Total-p38_Annotated_Replicates#1-2.jpg]

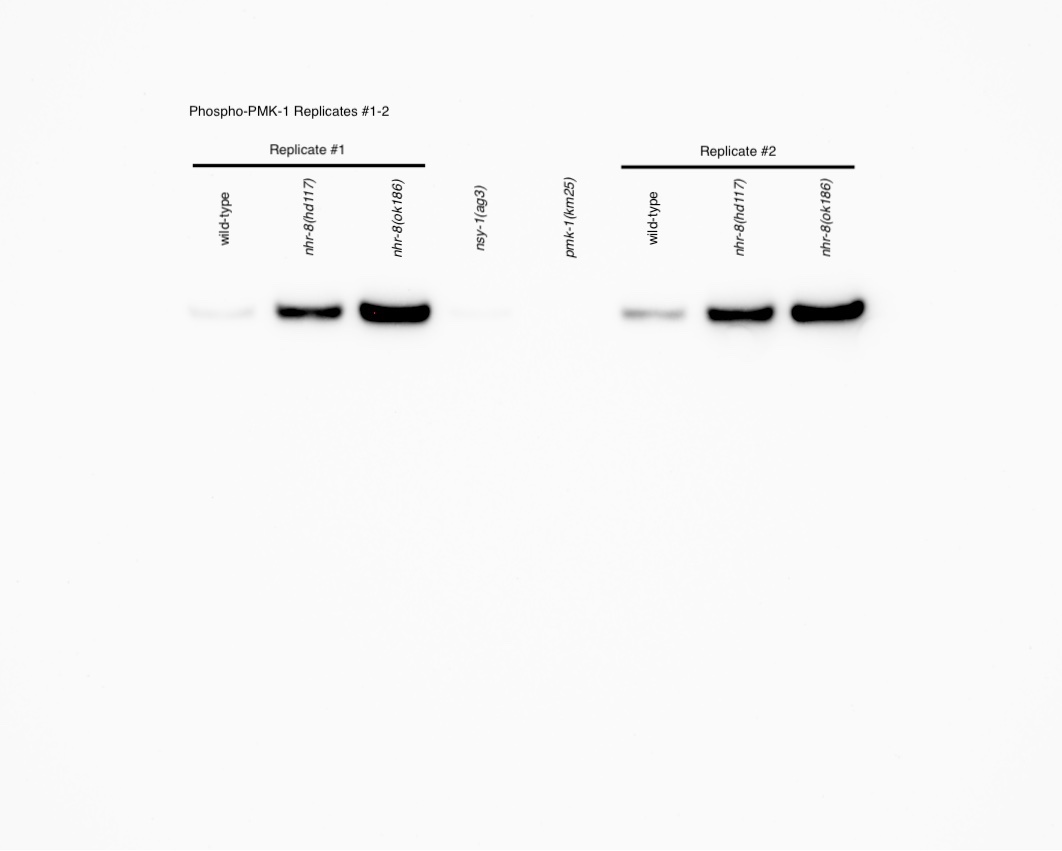

Supplement: Source data 1. [file elife-74206-data1.zip › Raw and annotated gel and blot images 2 of 2/Fig. 4E phospho-p38_Annotated_Replicates#1-2.jpg]

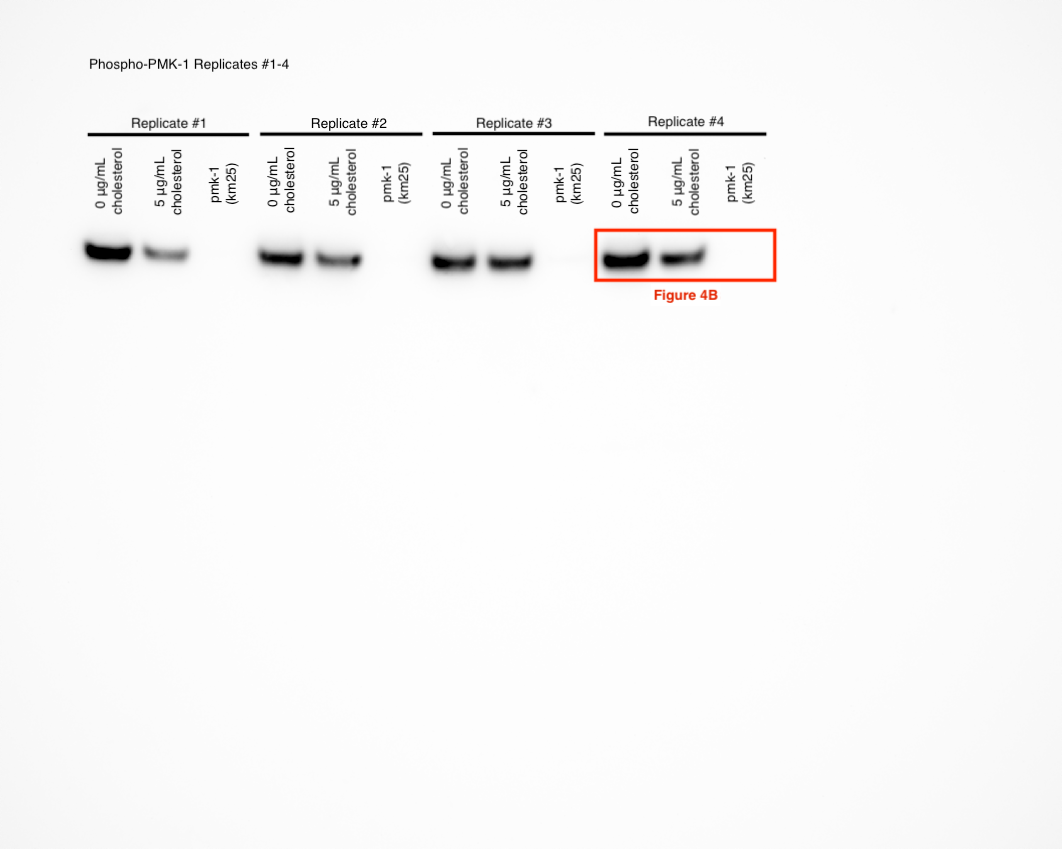

Supplement: Source data 1. [file elife-74206-data1.zip › Raw and annotated gel and blot images 2 of 2/Fig. 4B phospho-p38_Annotated_Replicates#1-4.tif]

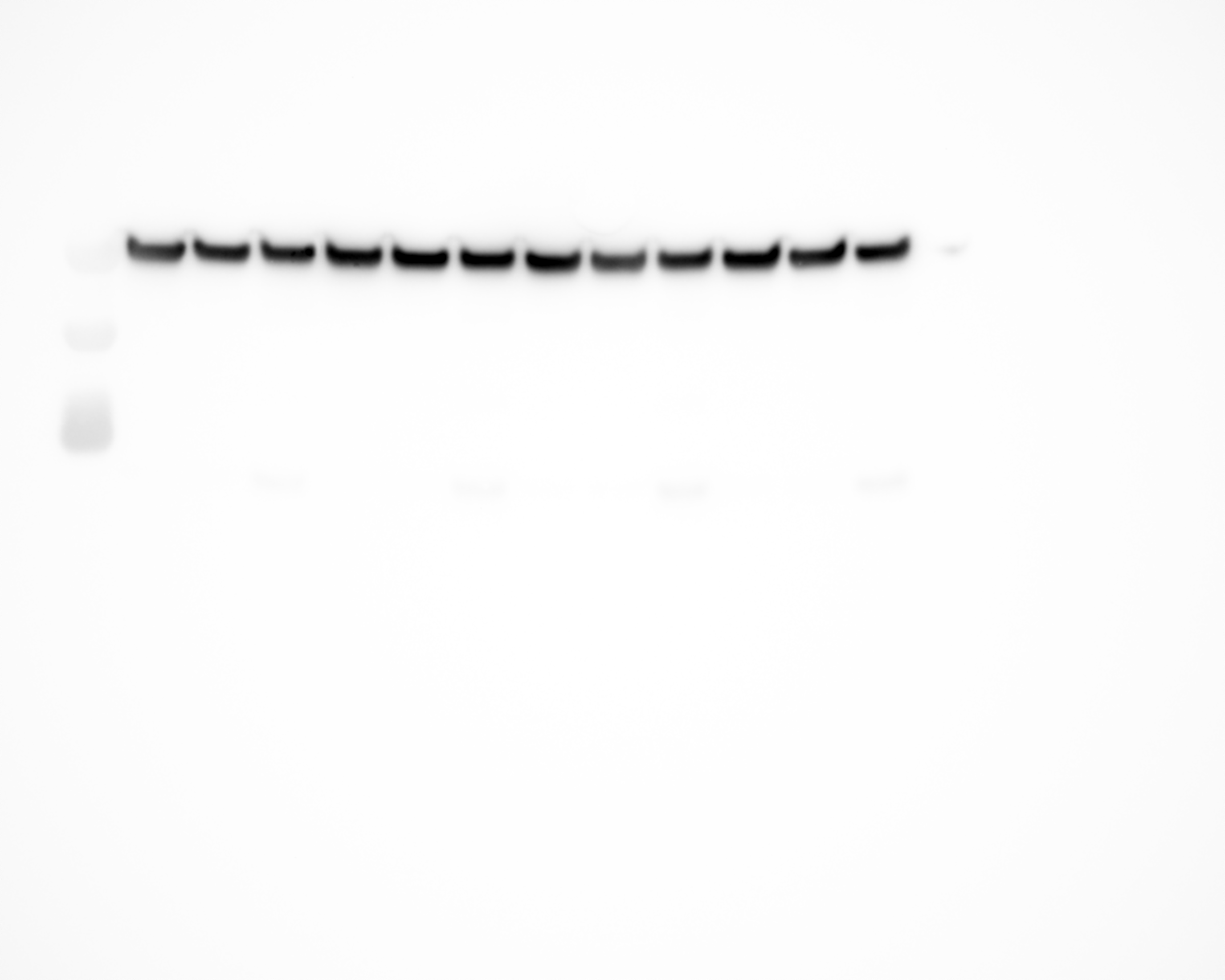

Supplement: Source data 1. [file elife-74206-data1.zip › Raw and annotated gel and blot images 2 of 2/Fig. 4B alpha_tubulin_raw_Replicates#1-4.tif]

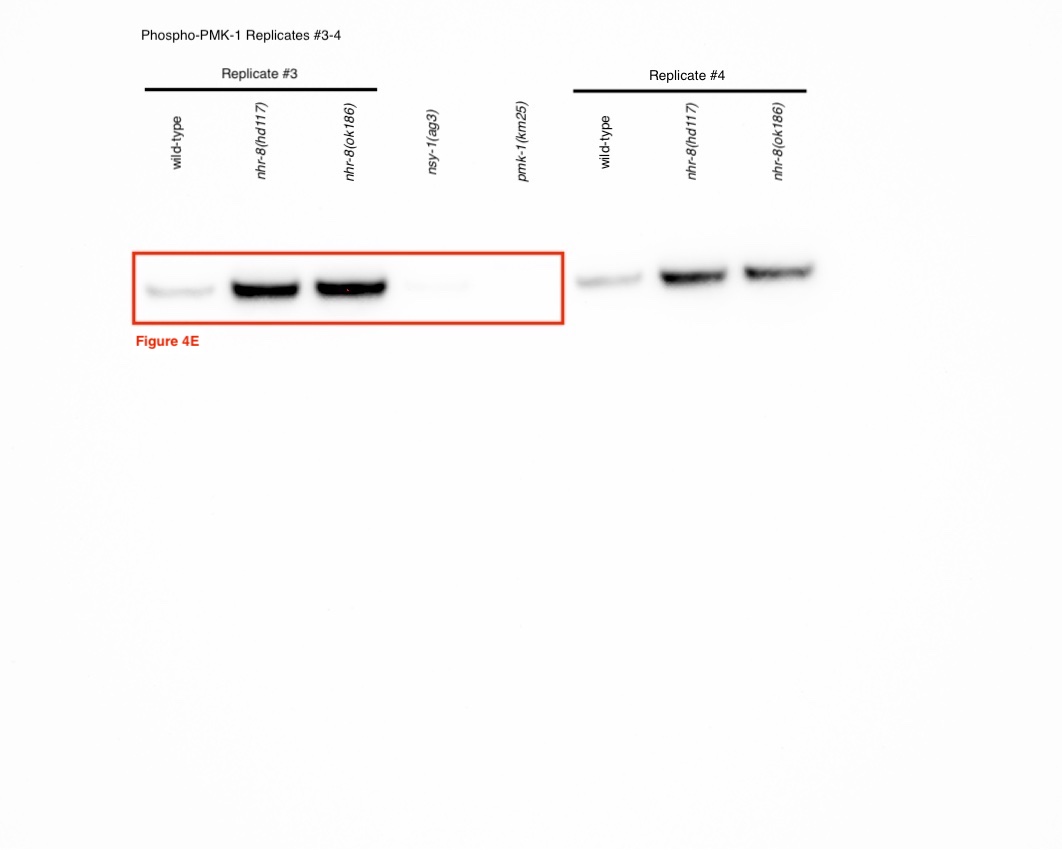

Supplement: Source data 1. [file elife-74206-data1.zip › Raw and annotated gel and blot images 2 of 2/Fig. 4E phospho-p38_Annotated_Replicates#3-4.jpg]

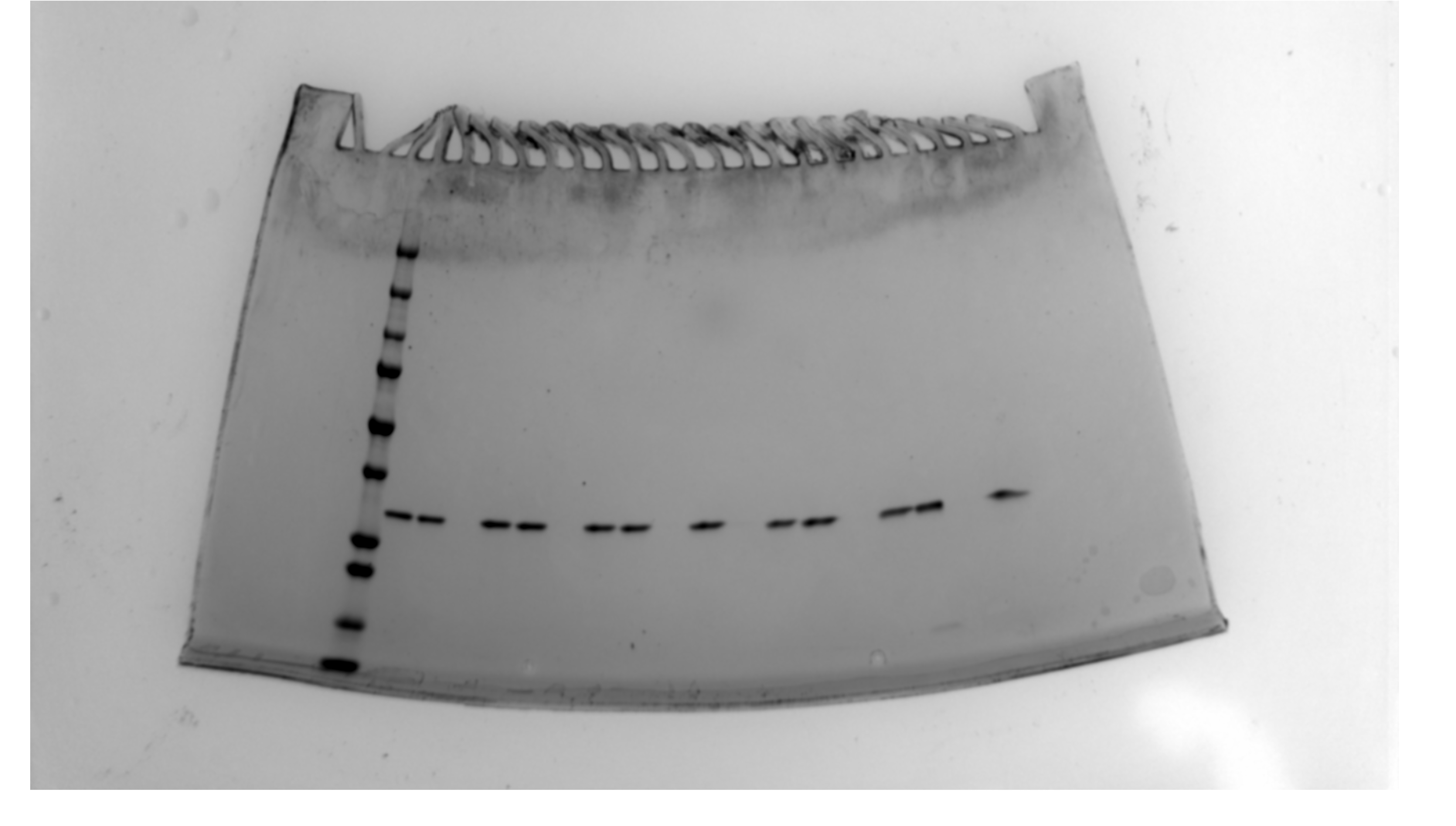

Supplement: Source data 1. [file elife-74206-data1.zip › Raw and annotated gel and blot images 2 of 2/Fig. 2 - figure supplement 1G_raw.png]

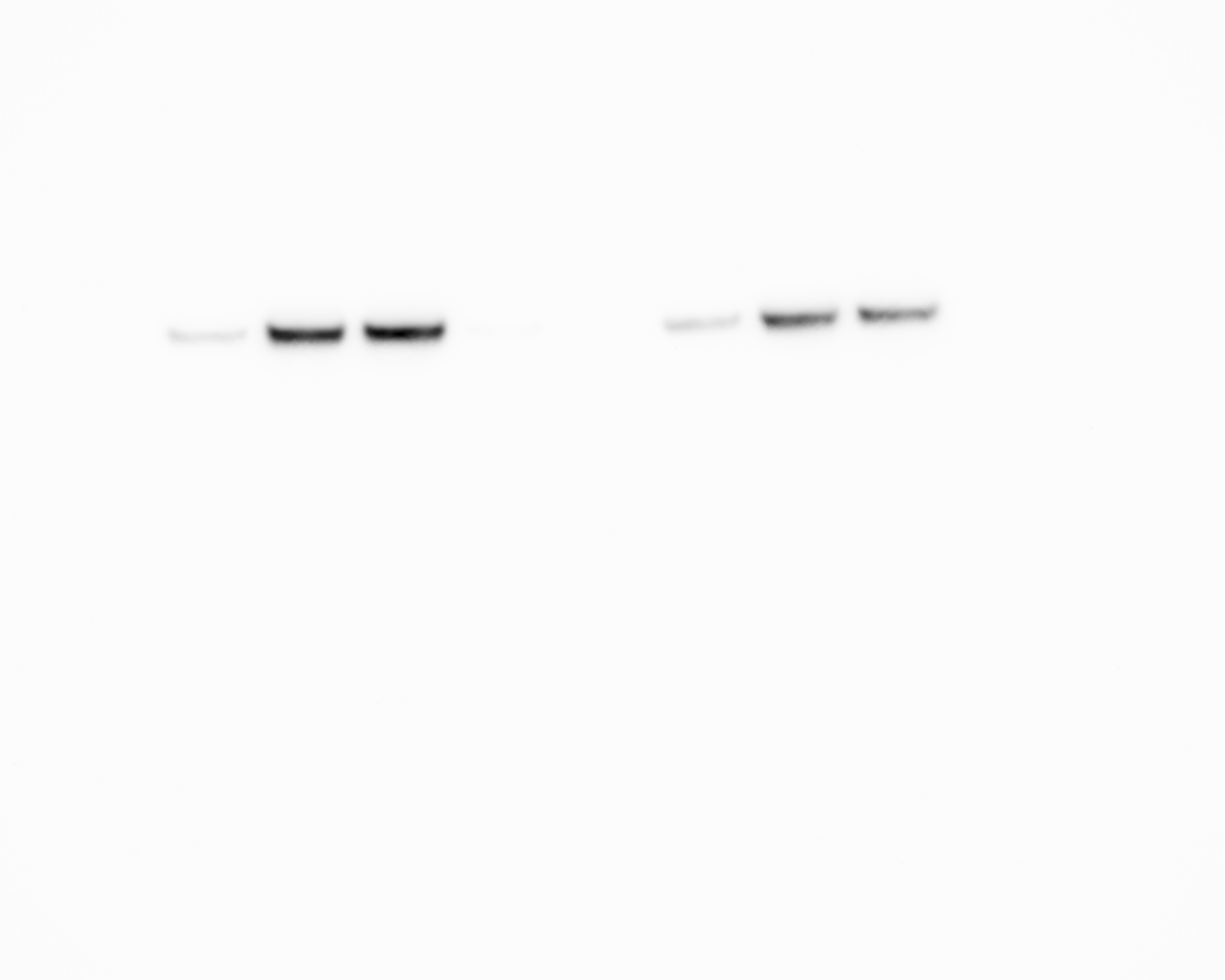

Supplement: Source data 1. [file elife-74206-data1.zip › Raw and annotated gel and blot images 2 of 2/Fig. 4E phospho-p38_raw_Replicates#3-4.tif]

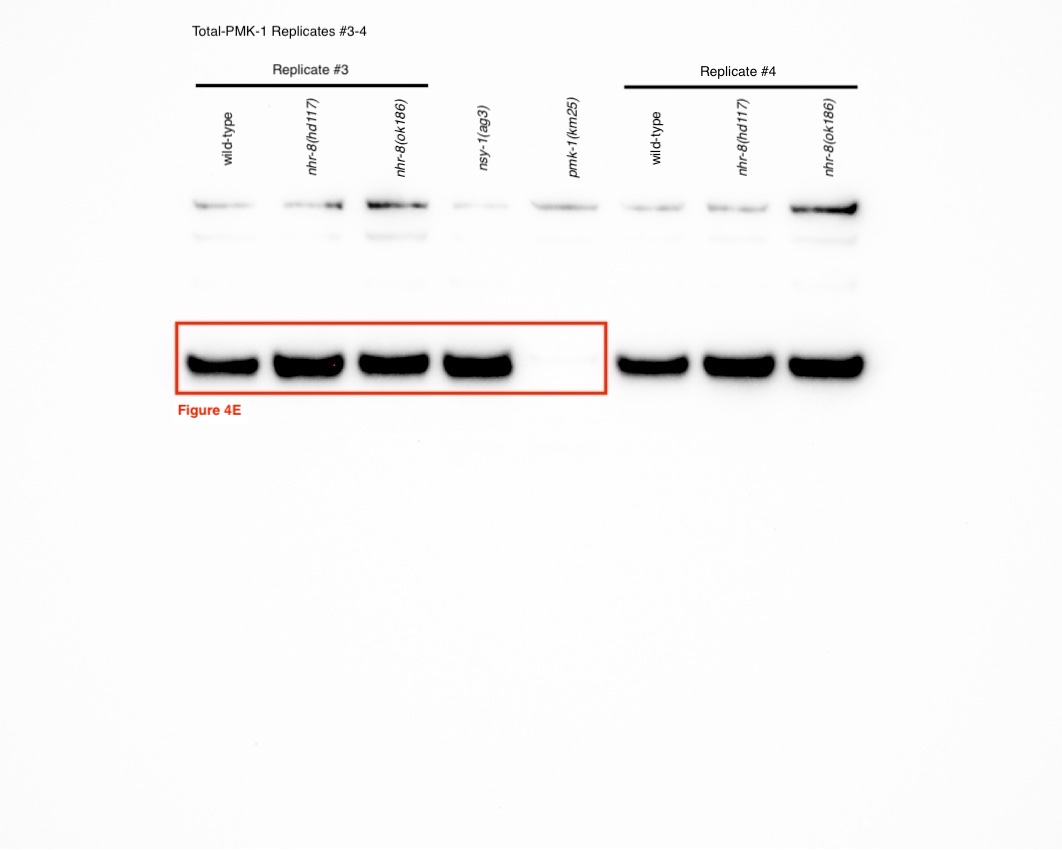

Supplement: Source data 1. [file elife-74206-data1.zip › Raw and annotated gel and blot images 2 of 2/Fig. 4E Total-p38_Annotated_Replicates#3-4.jpg]

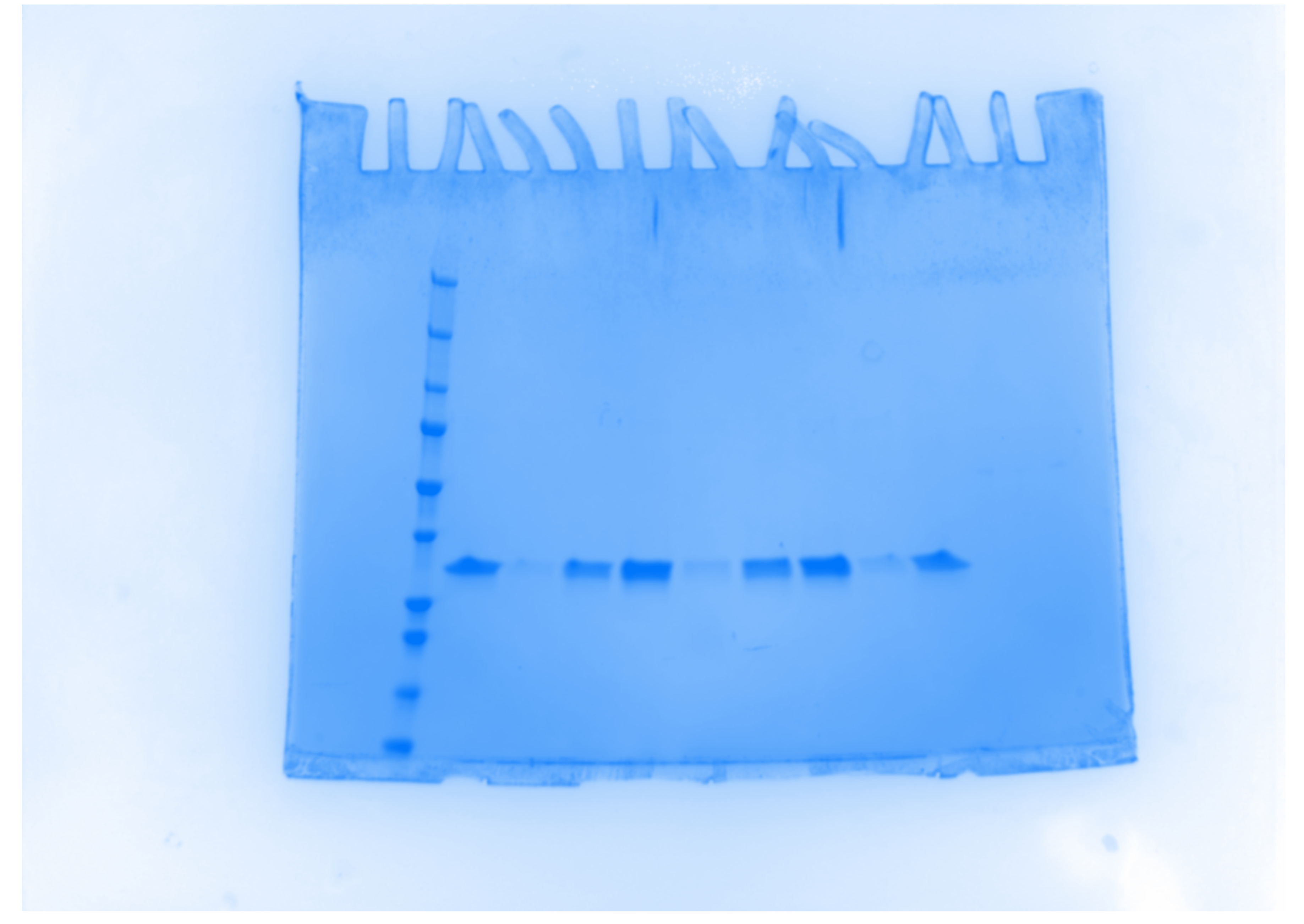

Supplement: Source data 1. [file elife-74206-data1.zip › Raw and annotated gel and blot images 2 of 2/Fig. 2 - figure supplement 2A_Citrate_raw.png]

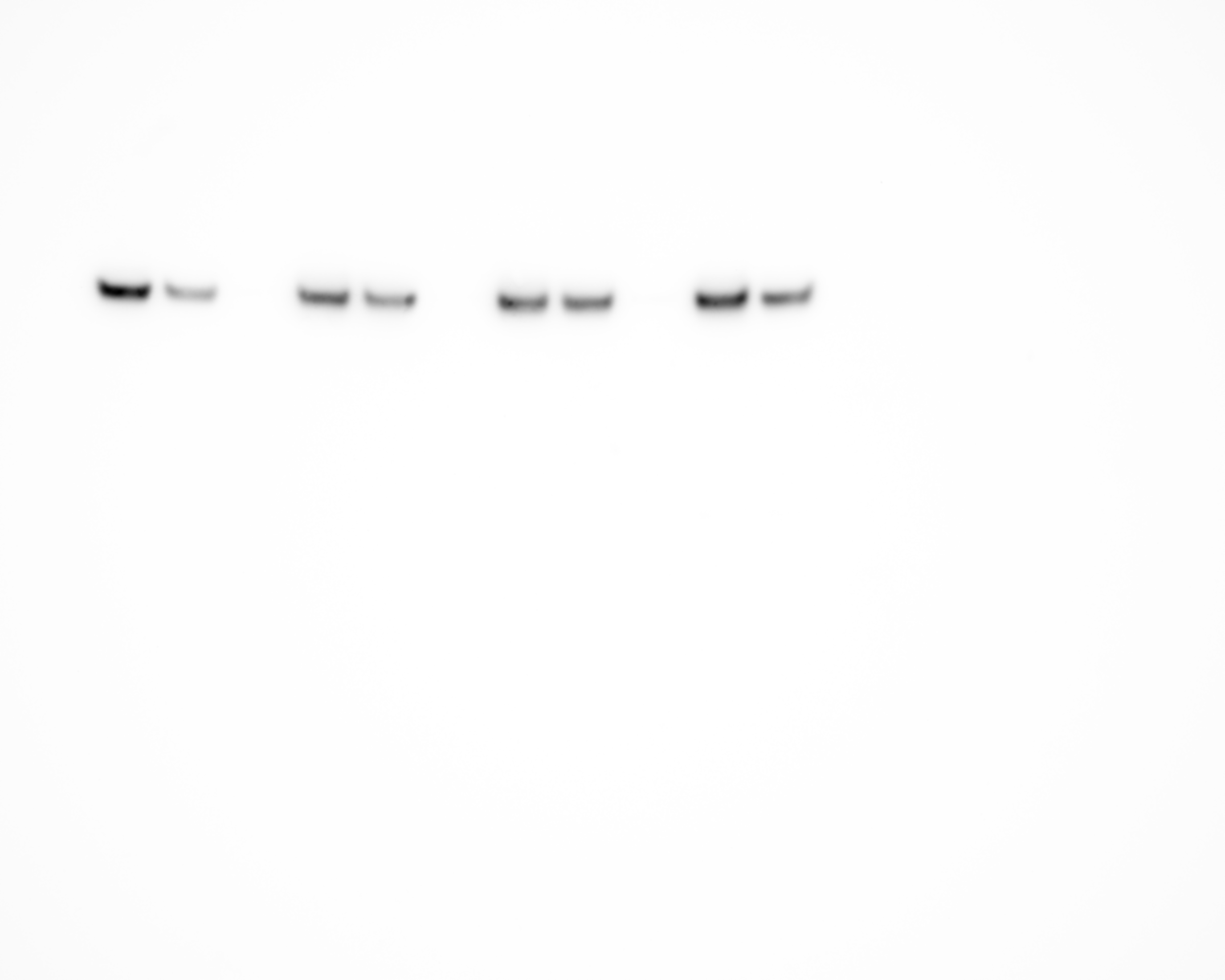

Supplement: Source data 1. [file elife-74206-data1.zip › Raw and annotated gel and blot images 2 of 2/Fig. 4B phospho-p38_raw_Replicates#1-4.tif]

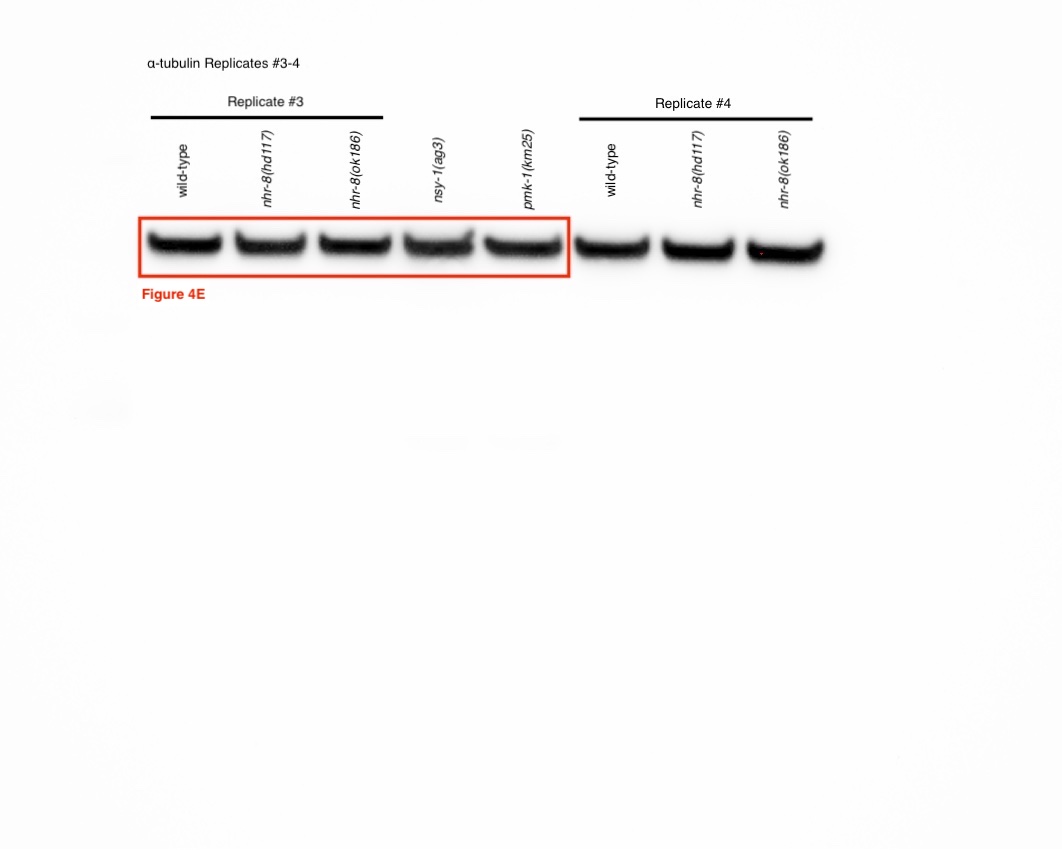

Supplement: Source data 1. [file elife-74206-data1.zip › Raw and annotated gel and blot images 2 of 2/Fig. 4E Alpha tubulin_Annotated_Replicates#3-4.jpg]

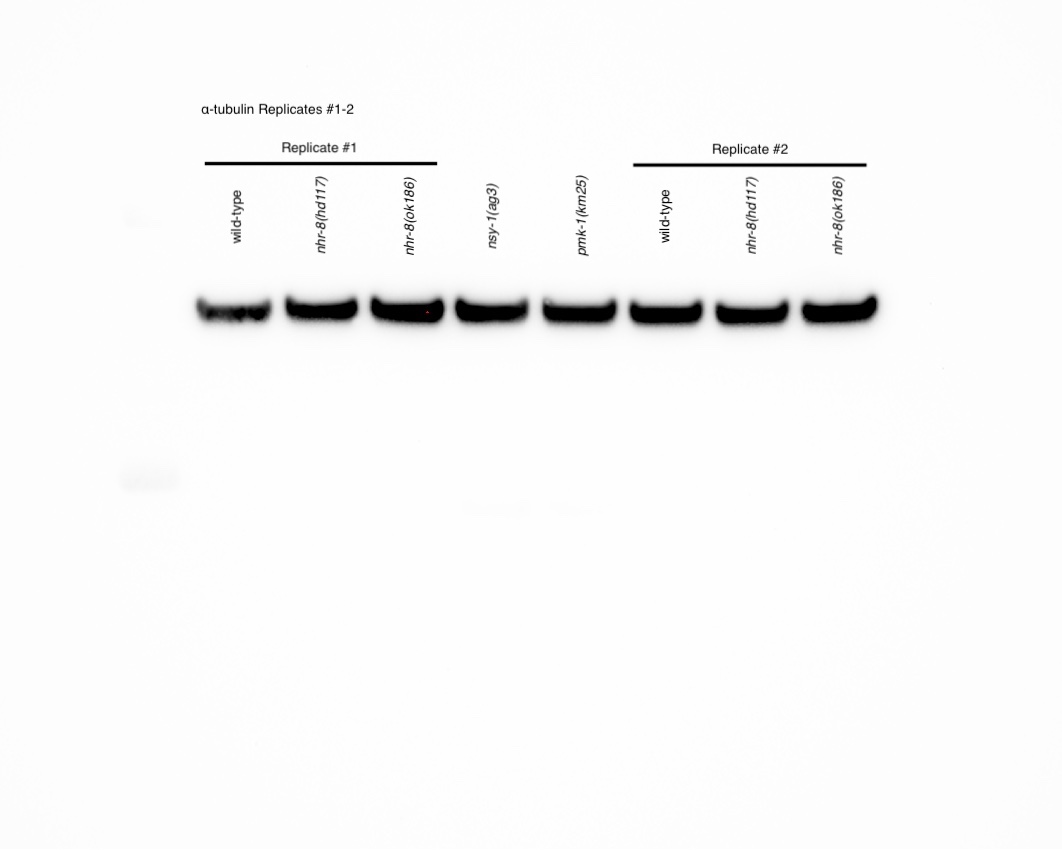

Supplement: Source data 1. [file elife-74206-data1.zip › Raw and annotated gel and blot images 2 of 2/Fig. 4E Alpha tubulin_Annotated_Replicates#1-2.jpg]

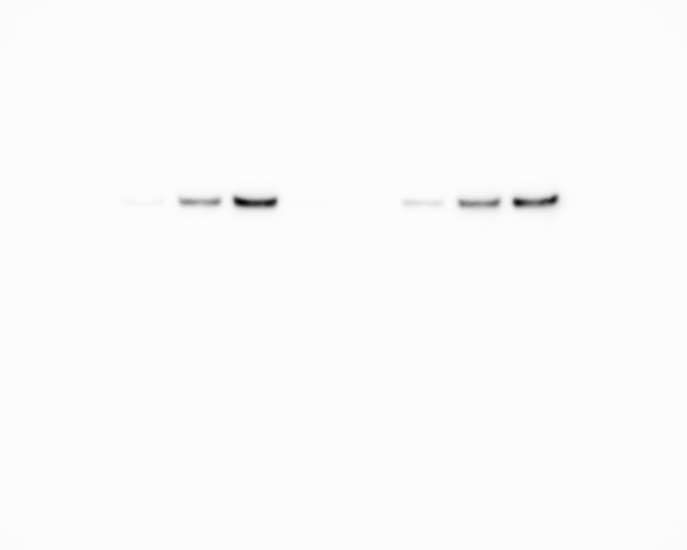

Supplement: Source data 1. [file elife-74206-data1.zip › Raw and annotated gel and blot images 2 of 2/Fig. 4E phospho-p38_raw_Replicates#1-2.jpg]

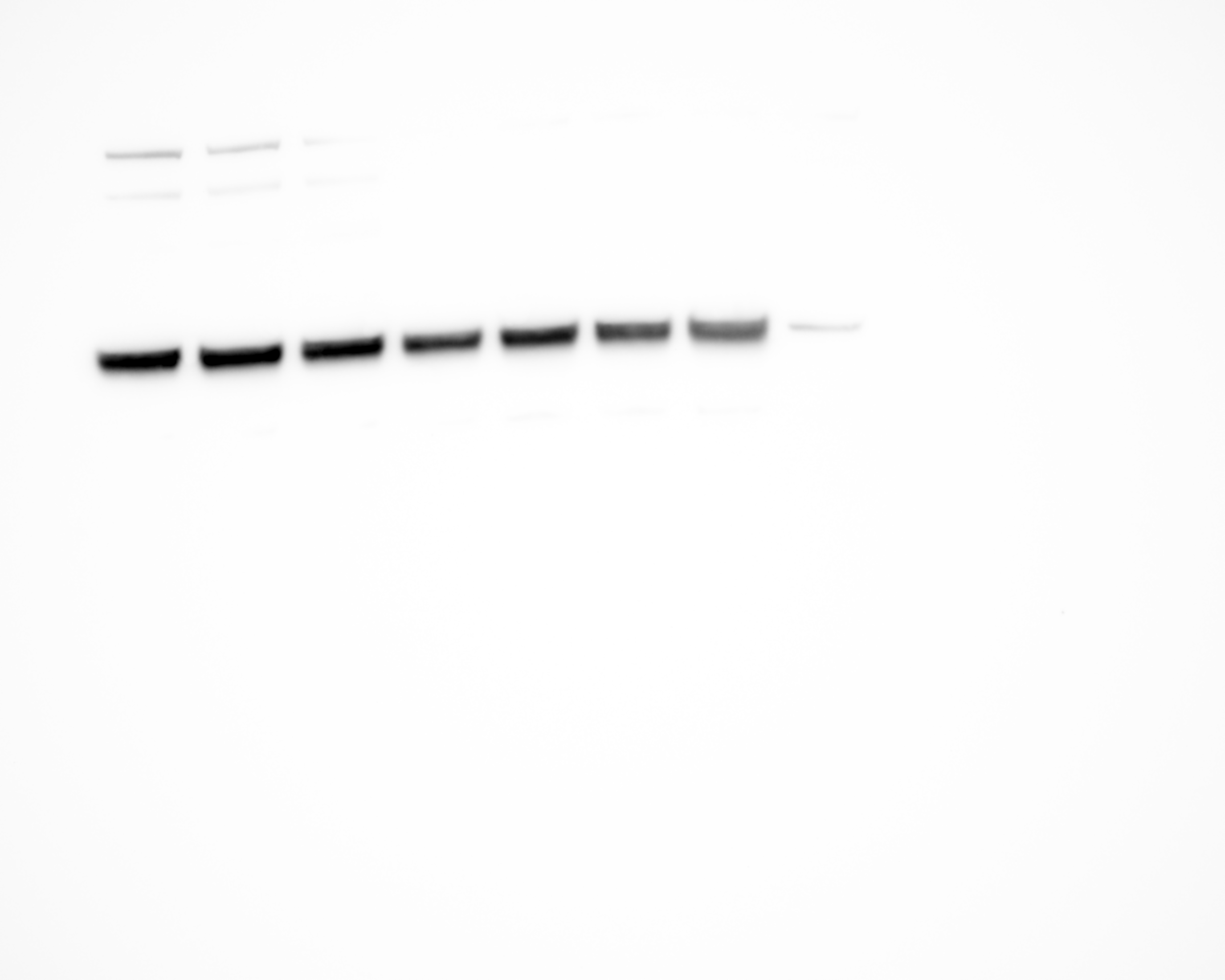

Supplement: Source data 2. [file elife-74206-data2.zip › Raw and annotated gel and blot images 1 of 2/Fig. 1E Total-p38_raw_Replicate#3.tif]

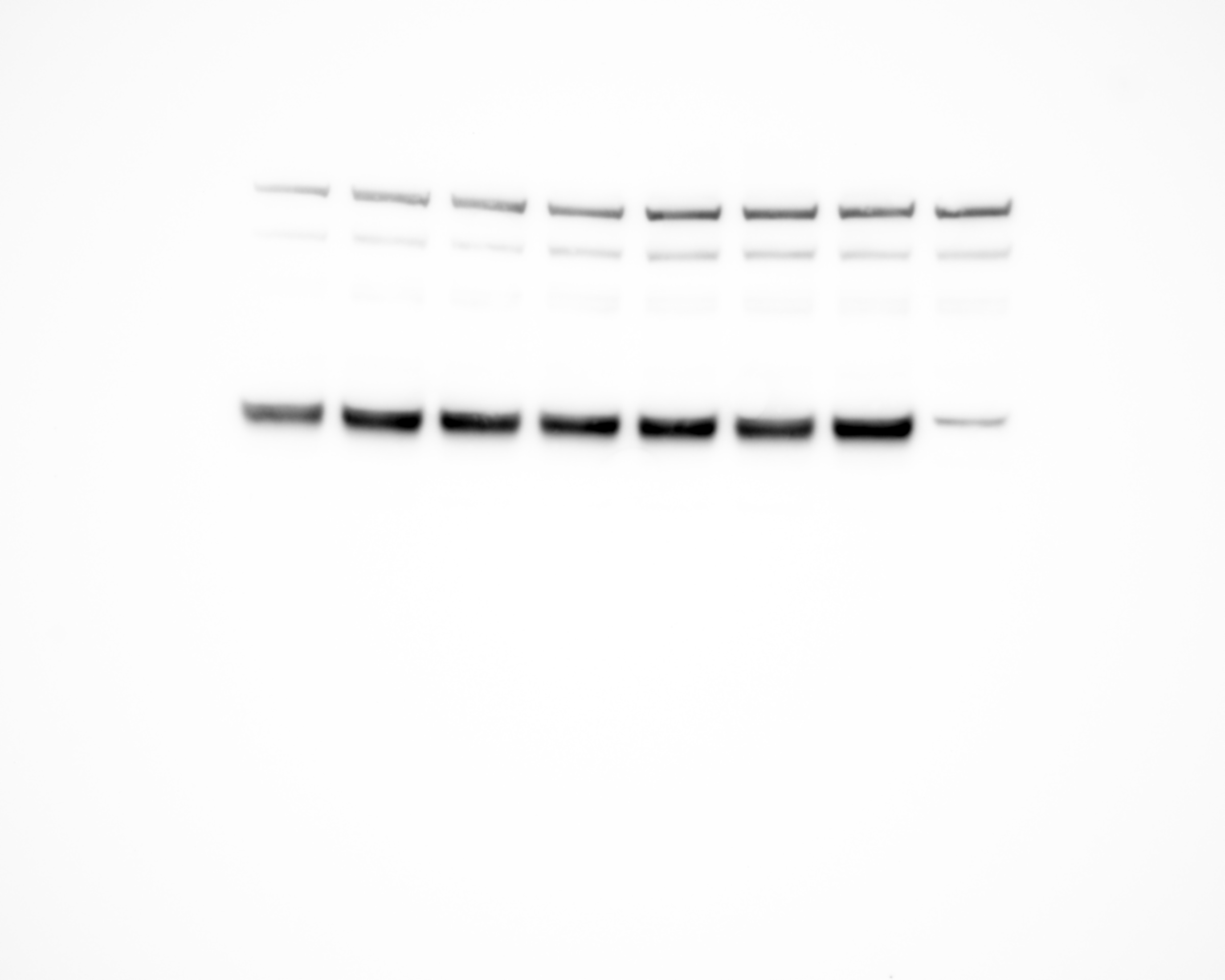

Supplement: Source data 2. [file elife-74206-data2.zip › Raw and annotated gel and blot images 1 of 2/Fig. 1E Total-p38_raw_Replicate#2.tif]

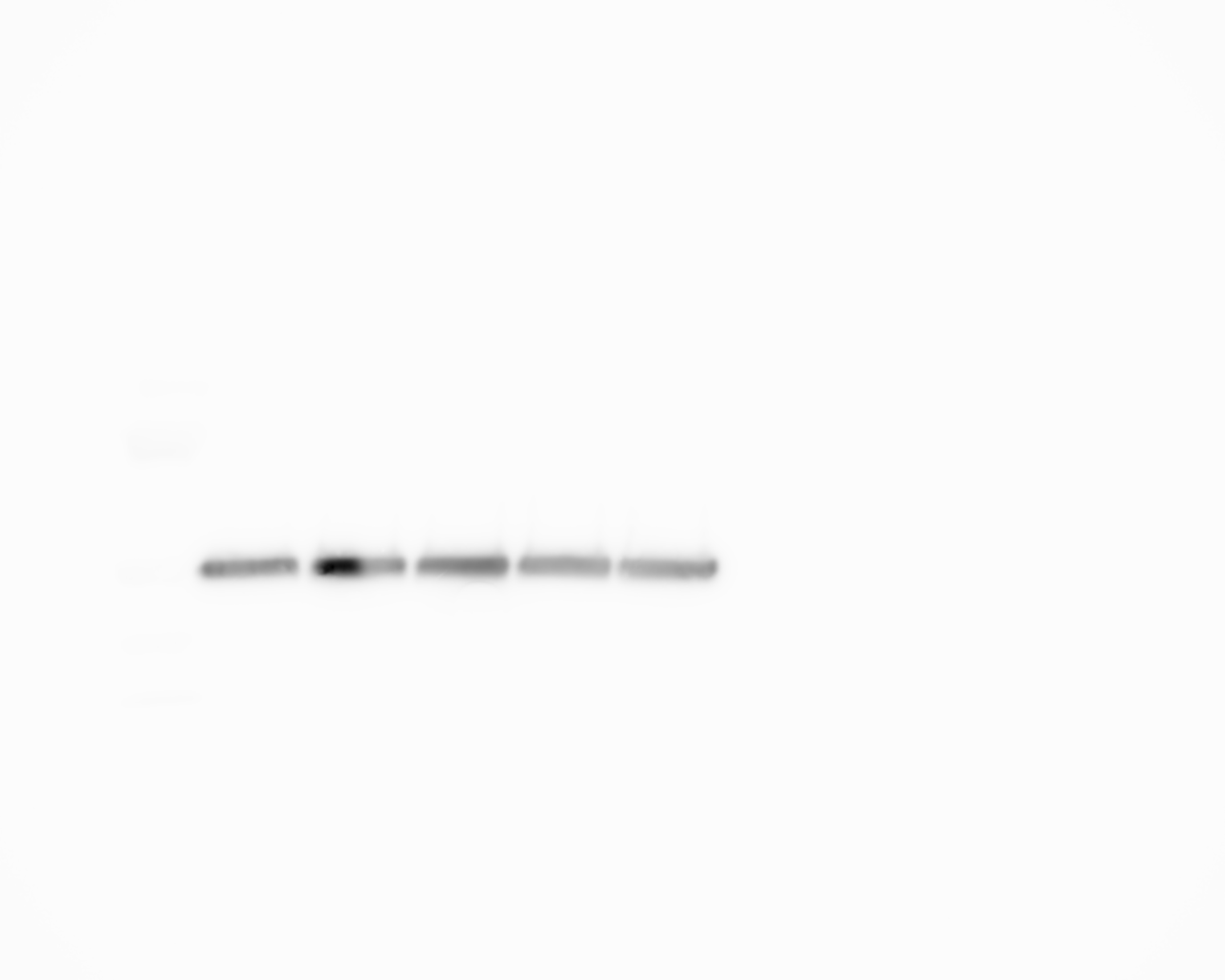

Supplement: Source data 2. [file elife-74206-data2.zip › Raw and annotated gel and blot images 1 of 2/Fig. 1 - figure supplement 1A Alpha tubulin_raw.tif]

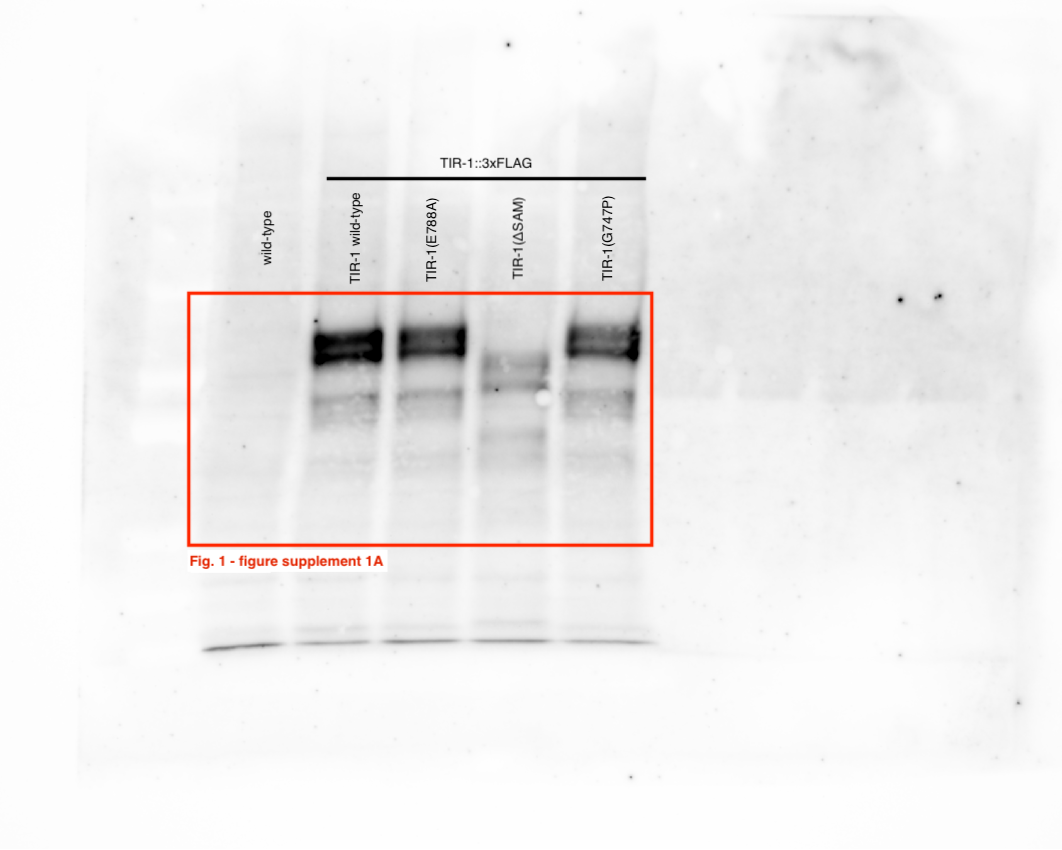

Supplement: Source data 2. [file elife-74206-data2.zip › Raw and annotated gel and blot images 1 of 2/Fig. 1 - figure supplement 1A FLAG_Annotated.tiff]

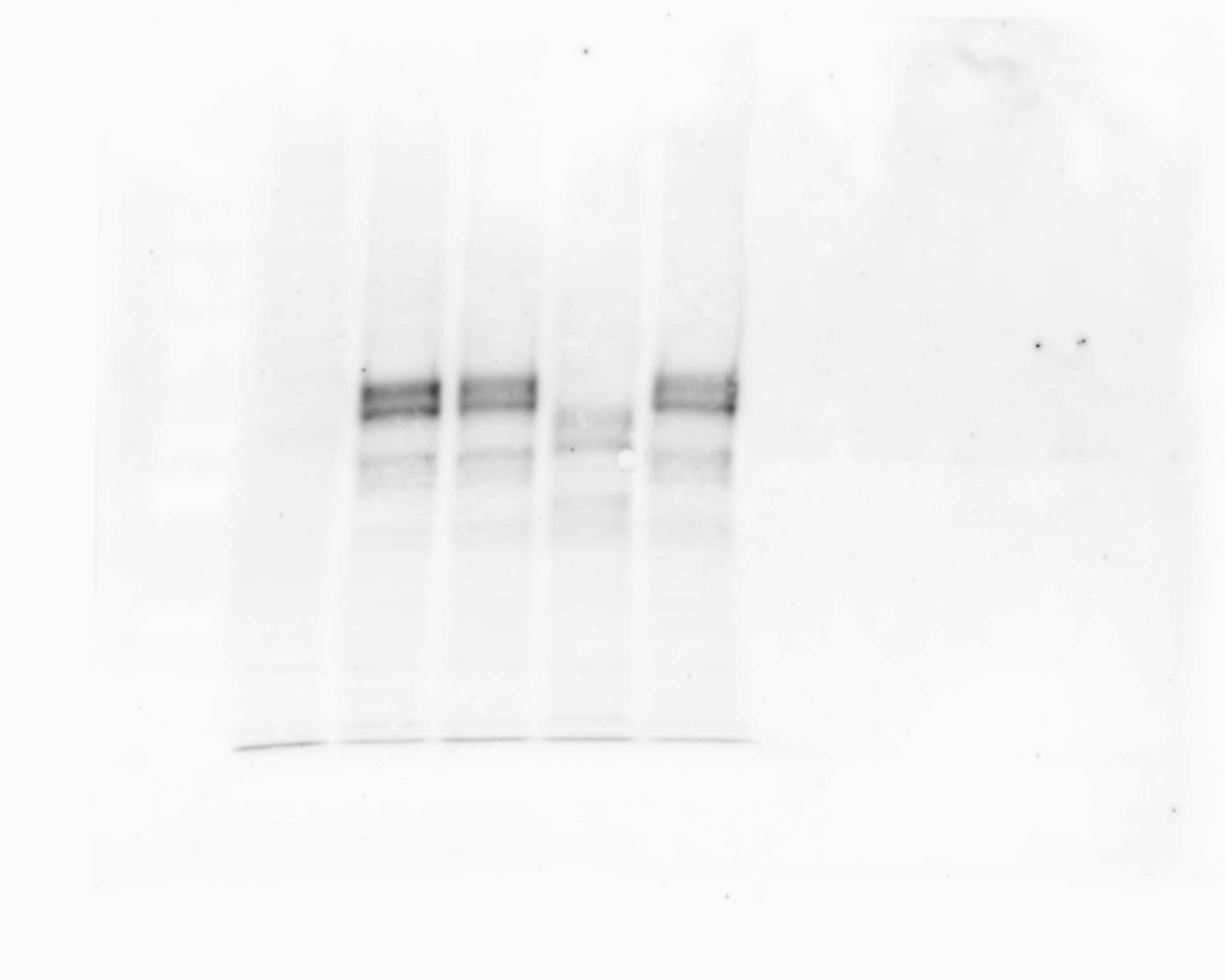

Supplement: Source data 2. [file elife-74206-data2.zip › Raw and annotated gel and blot images 1 of 2/Fig. 1 - figure supplement 1A FLAG_raw.tif]

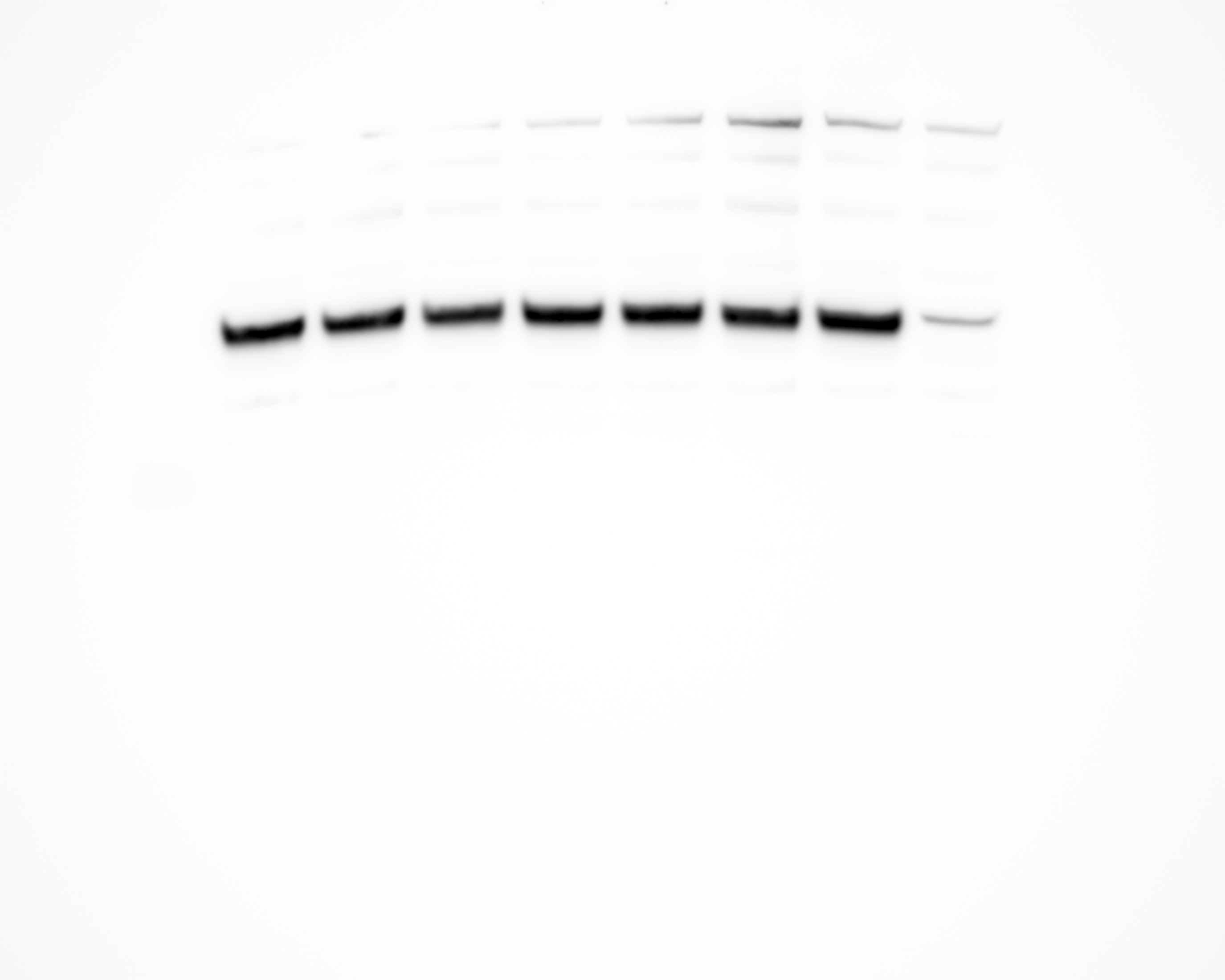

Supplement: Source data 2. [file elife-74206-data2.zip › Raw and annotated gel and blot images 1 of 2/Fig. 1E Total-p38_raw_Replicate#1.tif]

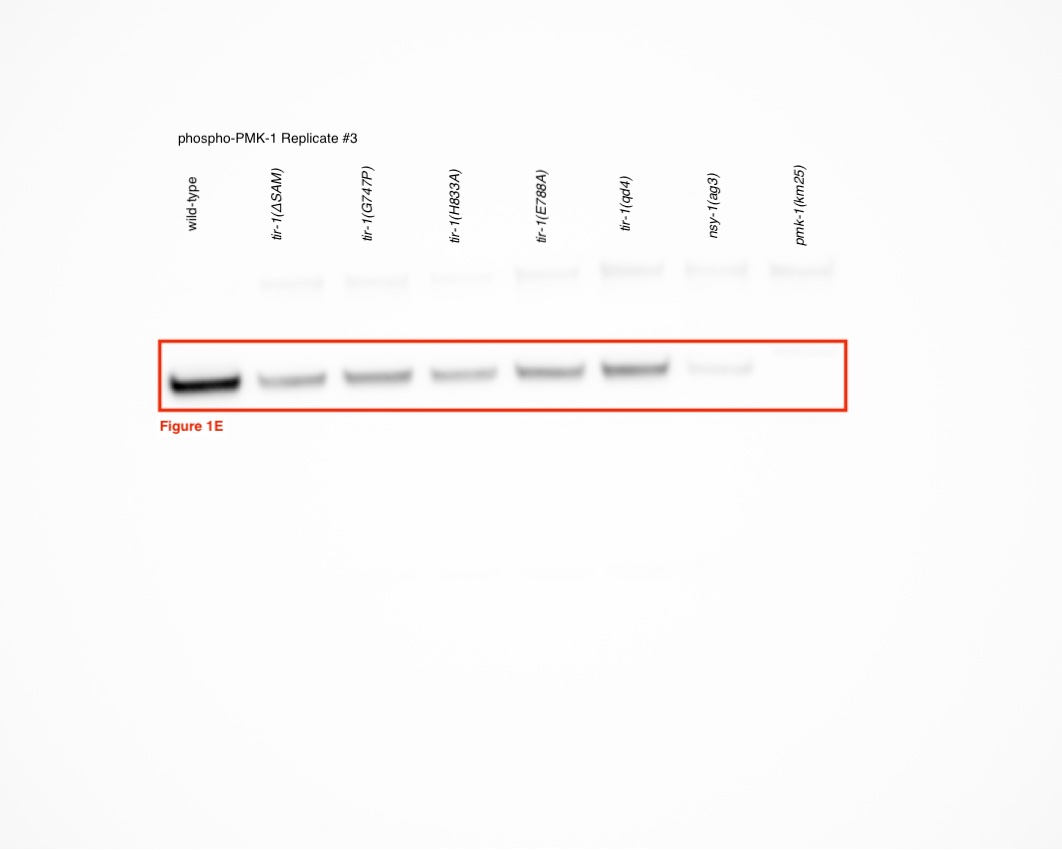

Supplement: Source data 2. [file elife-74206-data2.zip › Raw and annotated gel and blot images 1 of 2/Fig. 1E phospho-p38_Annotated_Replicate#3.jpg]

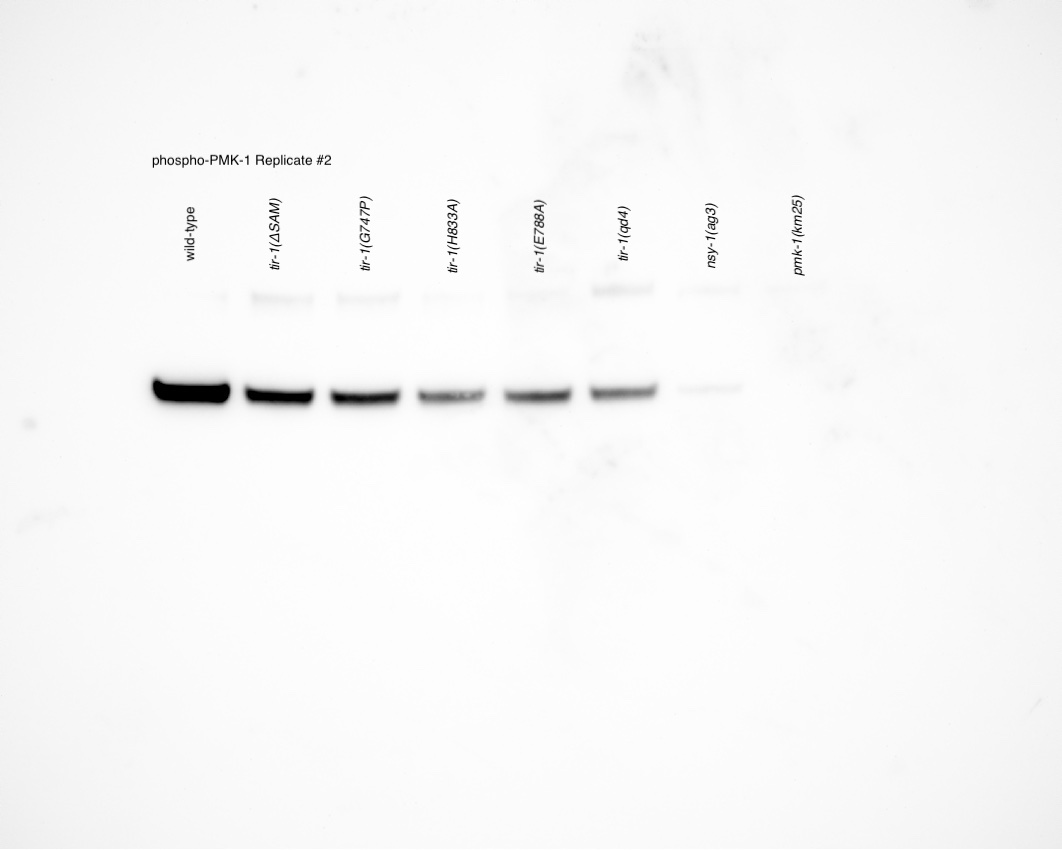

Supplement: Source data 2. [file elife-74206-data2.zip › Raw and annotated gel and blot images 1 of 2/Fig. 1E phospho-p38_Annotated_Replicate#2.jpg]

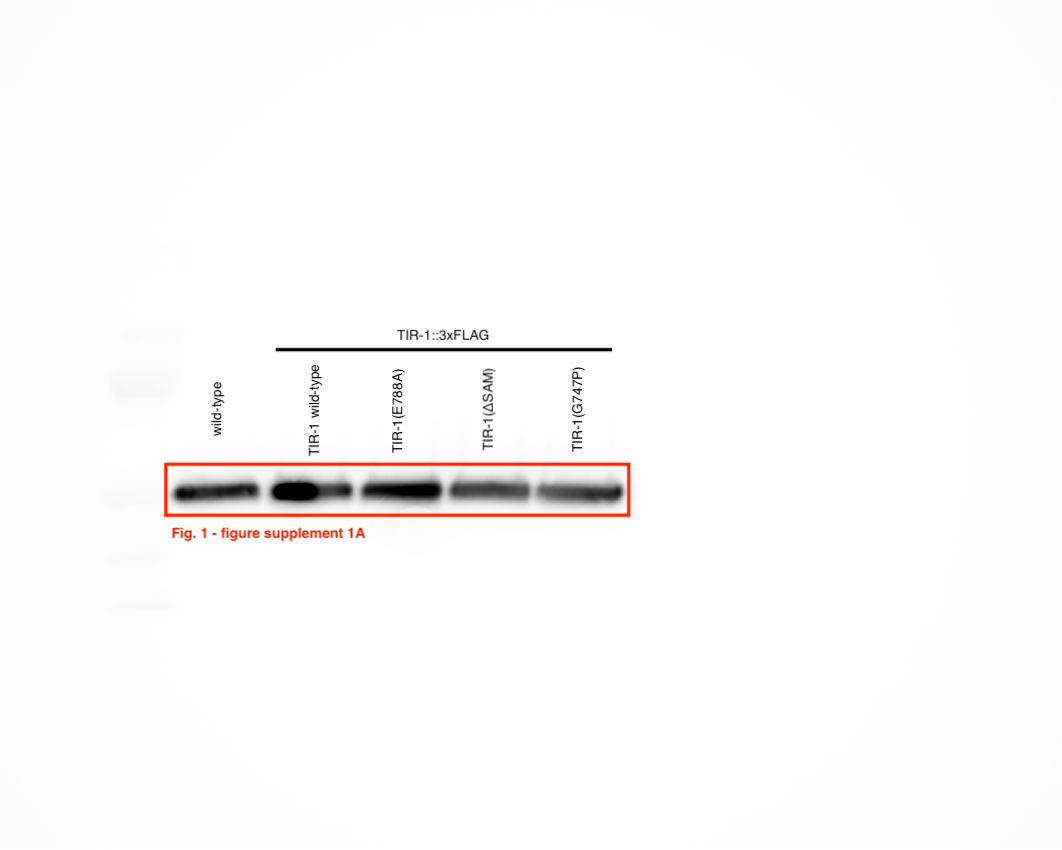

Supplement: Source data 2. [file elife-74206-data2.zip › Raw and annotated gel and blot images 1 of 2/Fig. 1 - figure supplement 1A Alpha tubulin_Annotated.tiff]

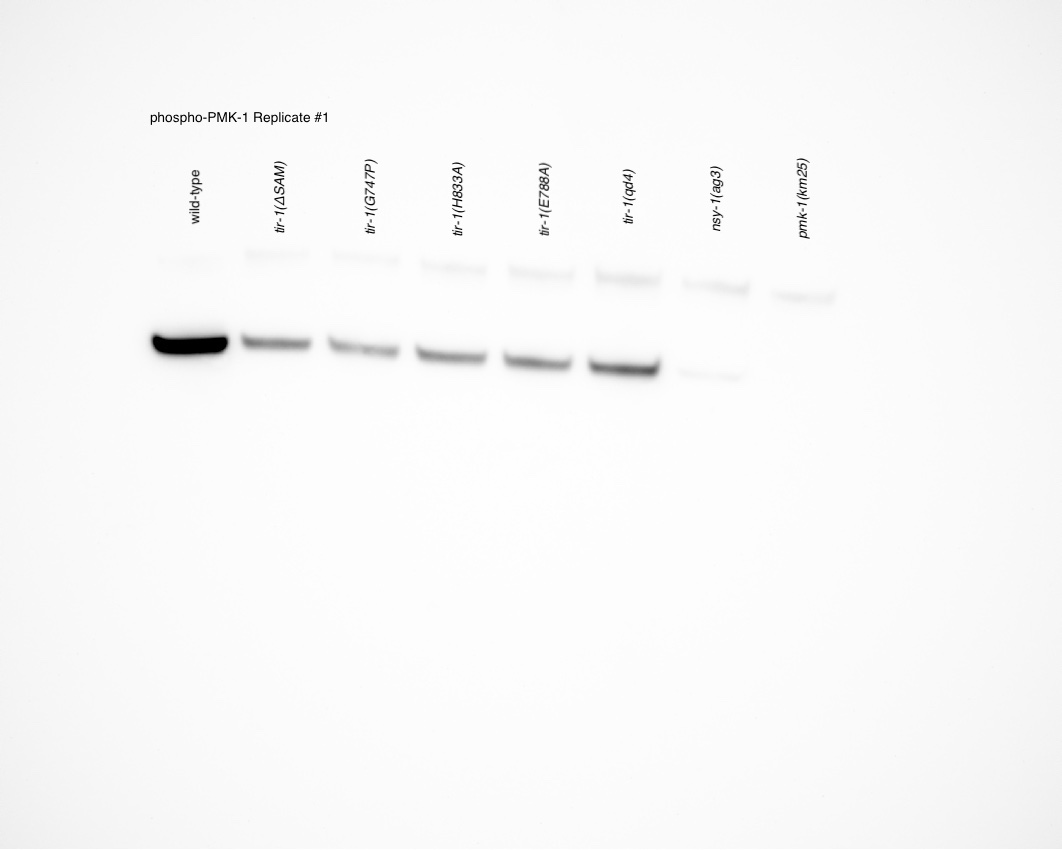

Supplement: Source data 2. [file elife-74206-data2.zip › Raw and annotated gel and blot images 1 of 2/Fig. 1E phospho-p38_Annotated_Replicate#1.jpg]

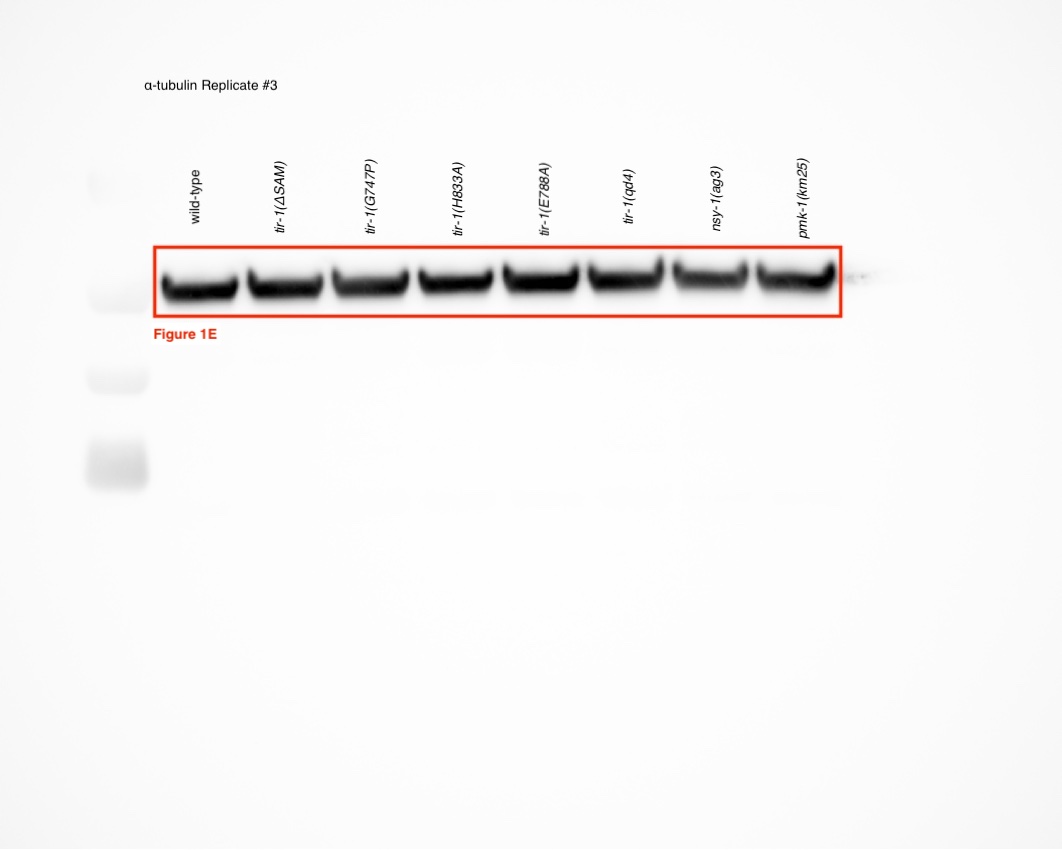

Supplement: Source data 2. [file elife-74206-data2.zip › Raw and annotated gel and blot images 1 of 2/Fig. 1E Alpha tubulin_Annotated_Replicate#3.jpg]

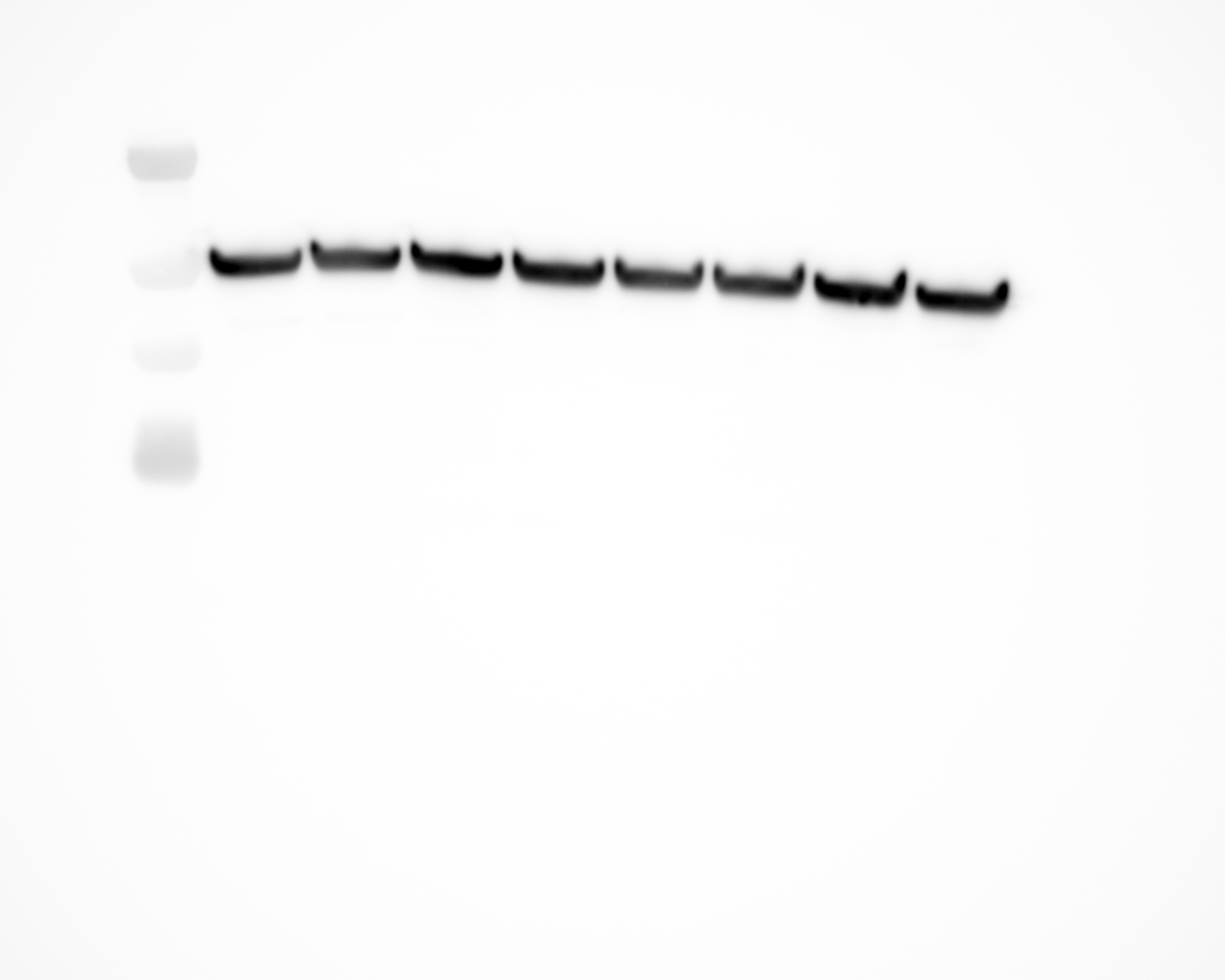

Supplement: Source data 2. [file elife-74206-data2.zip › Raw and annotated gel and blot images 1 of 2/Fig. 1E Alpha tubulin_raw_Replicate#1.tif]

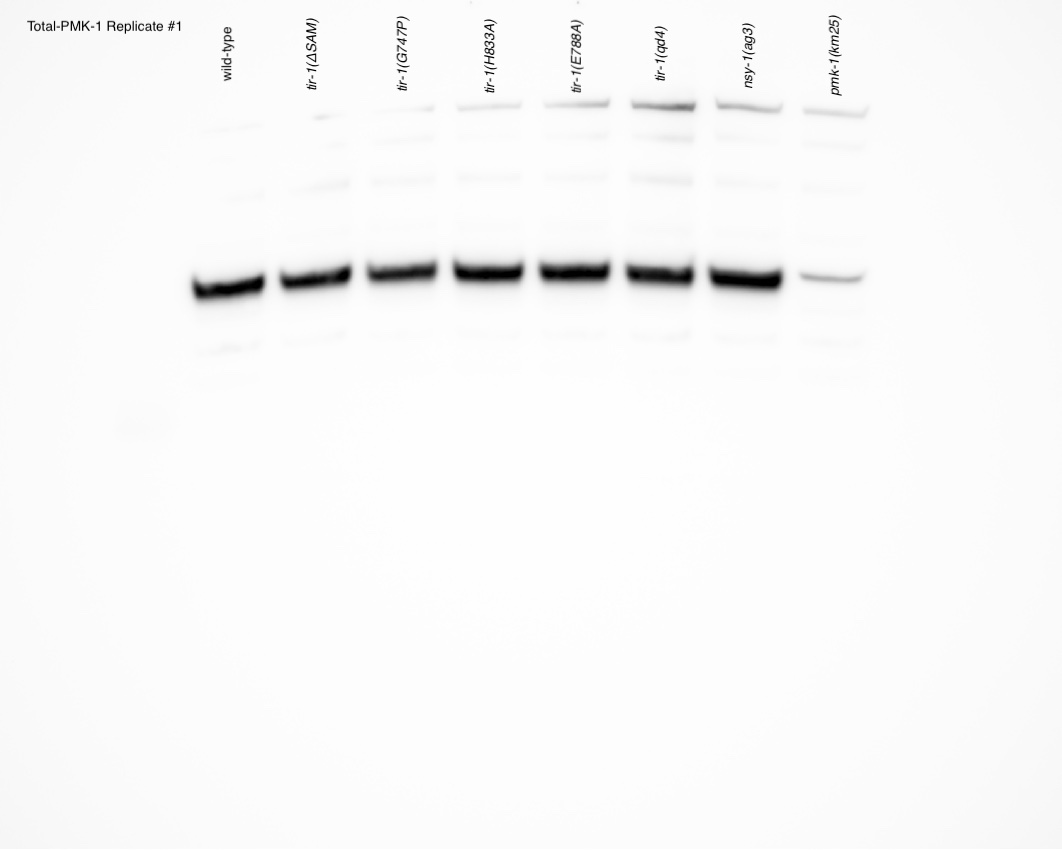

Supplement: Source data 2. [file elife-74206-data2.zip › Raw and annotated gel and blot images 1 of 2/Fig. 1E Total-p38_Annotated_Replicate#1.jpg]

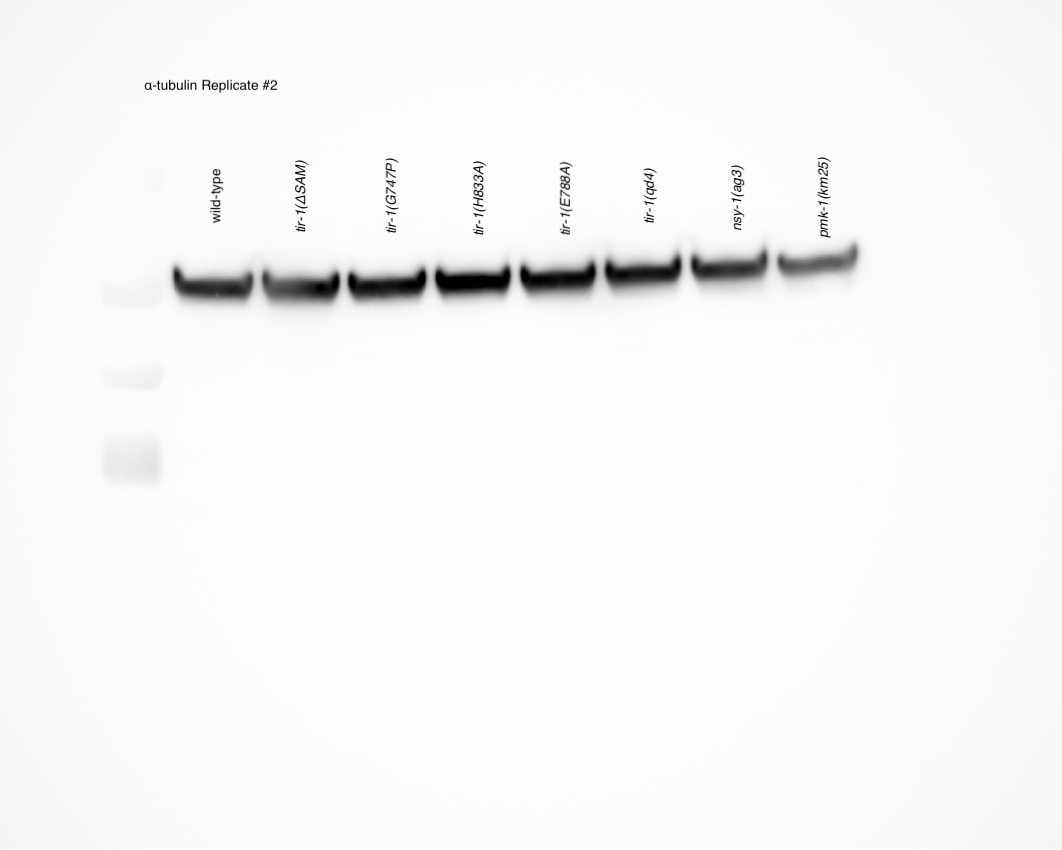

Supplement: Source data 2. [file elife-74206-data2.zip › Raw and annotated gel and blot images 1 of 2/Fig. 1E Alpha tubulin_Annotated_Replicate#2.jpg]

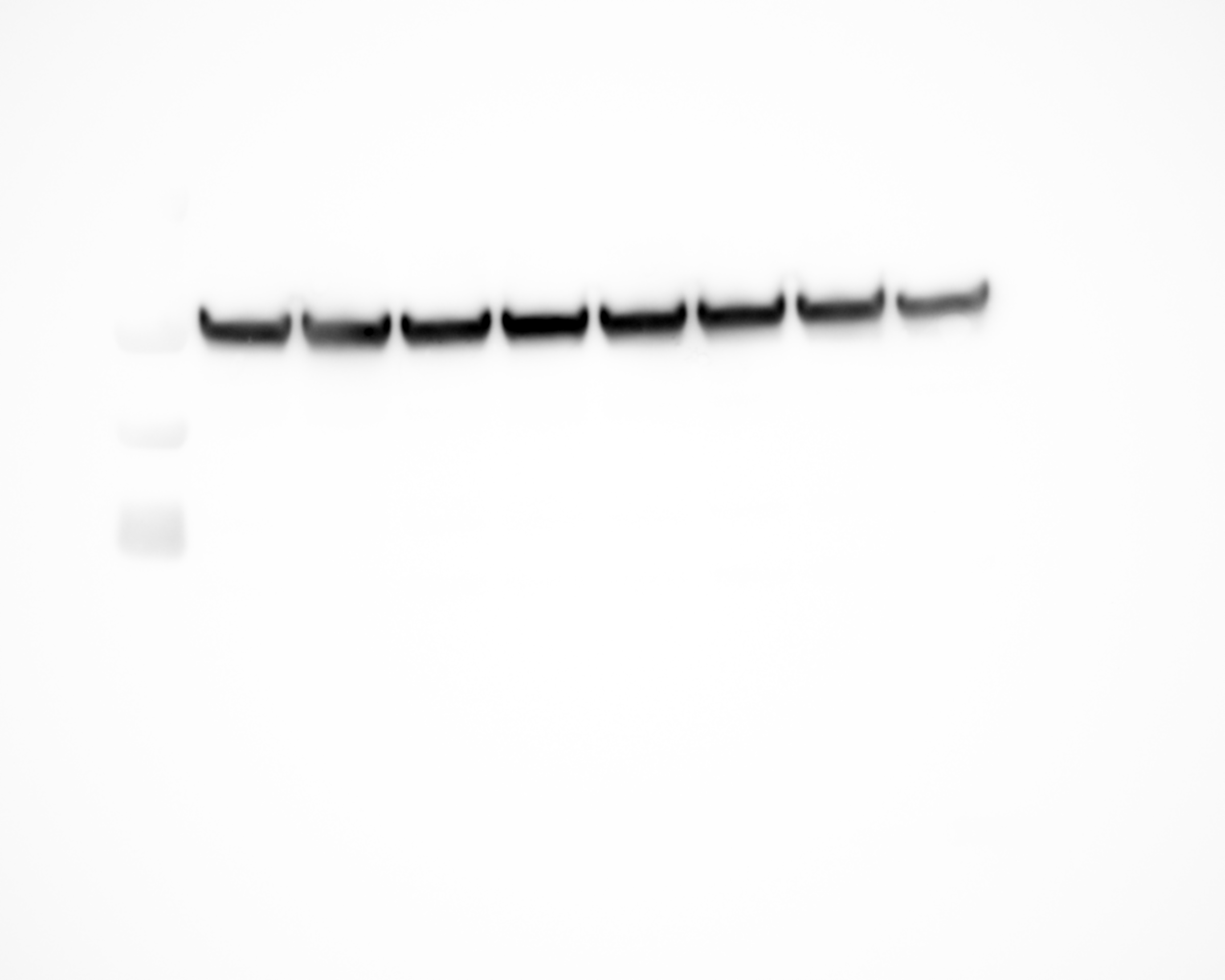

Supplement: Source data 2. [file elife-74206-data2.zip › Raw and annotated gel and blot images 1 of 2/Fig. 1E Alpha tubulin_raw_Replicate#2.tif]

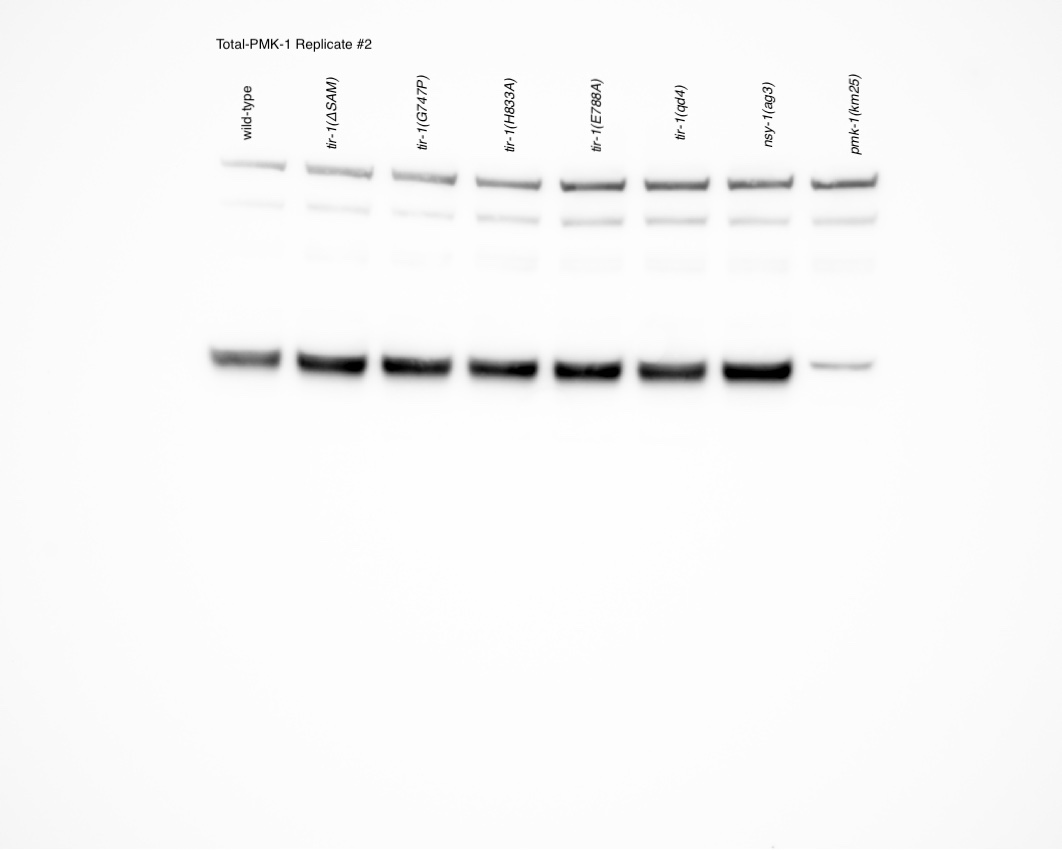

Supplement: Source data 2. [file elife-74206-data2.zip › Raw and annotated gel and blot images 1 of 2/Fig. 1E Total-p38_Annotated_Replicate#2.jpg]

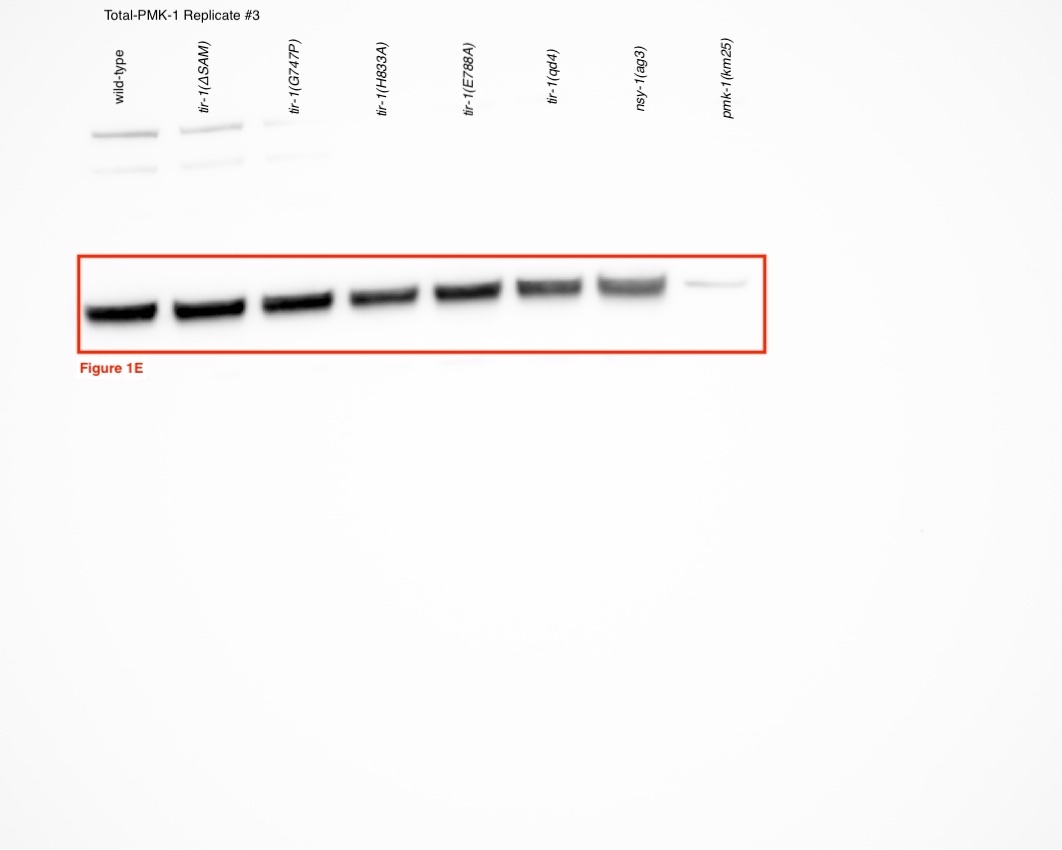

Supplement: Source data 2. [file elife-74206-data2.zip › Raw and annotated gel and blot images 1 of 2/Fig. 1E Total-p38_Annotated_Replicate#3.jpg]

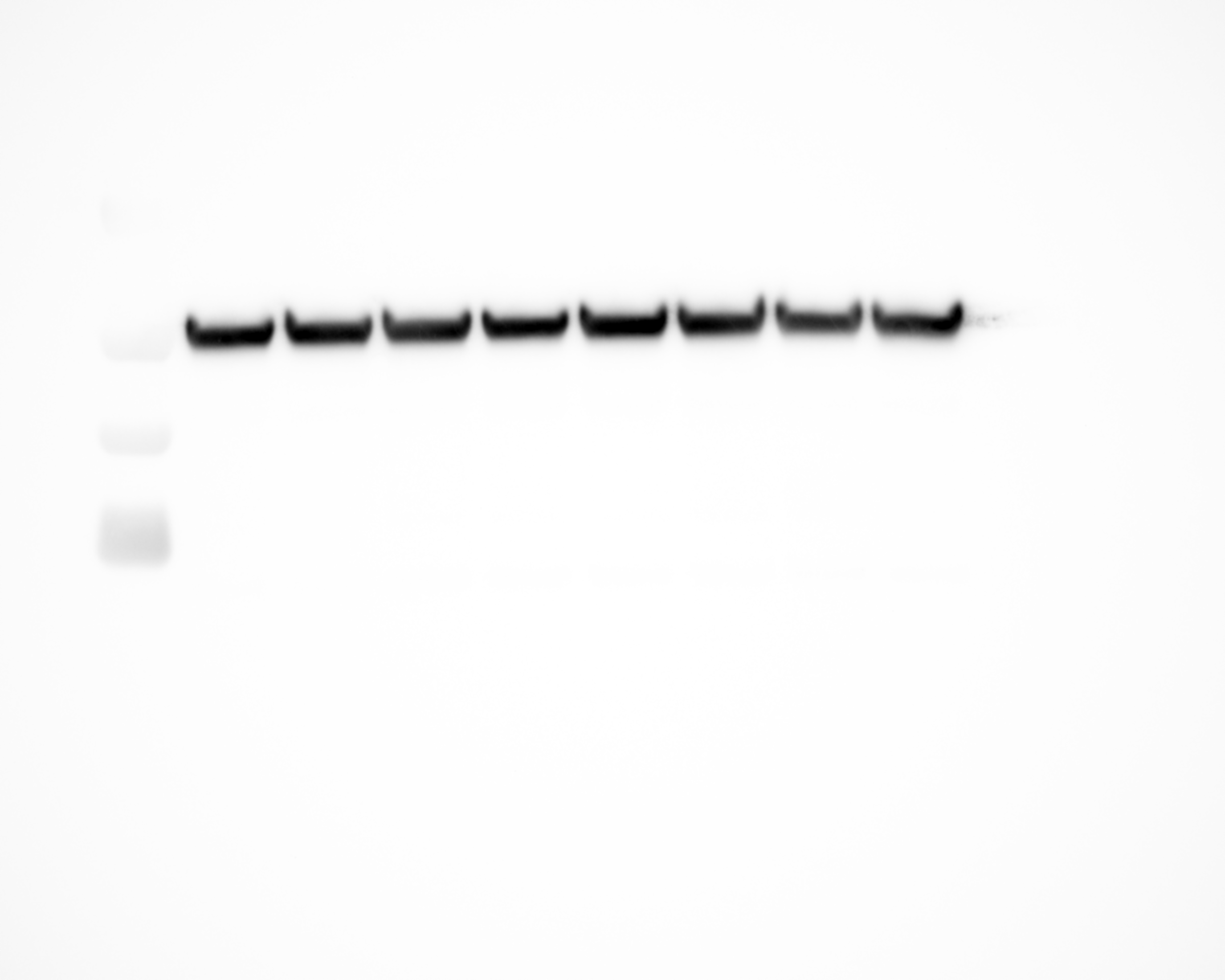

Supplement: Source data 2. [file elife-74206-data2.zip › Raw and annotated gel and blot images 1 of 2/Fig. 1E Alpha tubulin_raw_Replicate#3.tif]

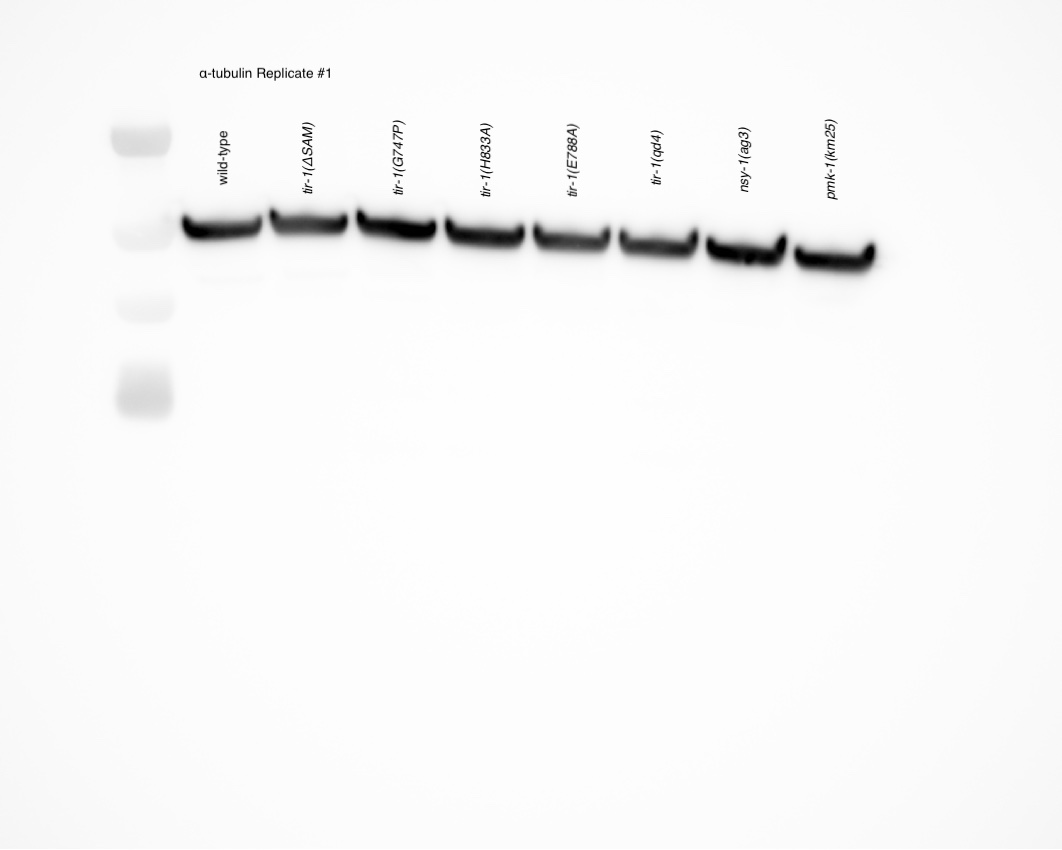

Supplement: Source data 2. [file elife-74206-data2.zip › Raw and annotated gel and blot images 1 of 2/Fig. 1E Alpha tubulin_Annotated_Replicate#1.jpg]

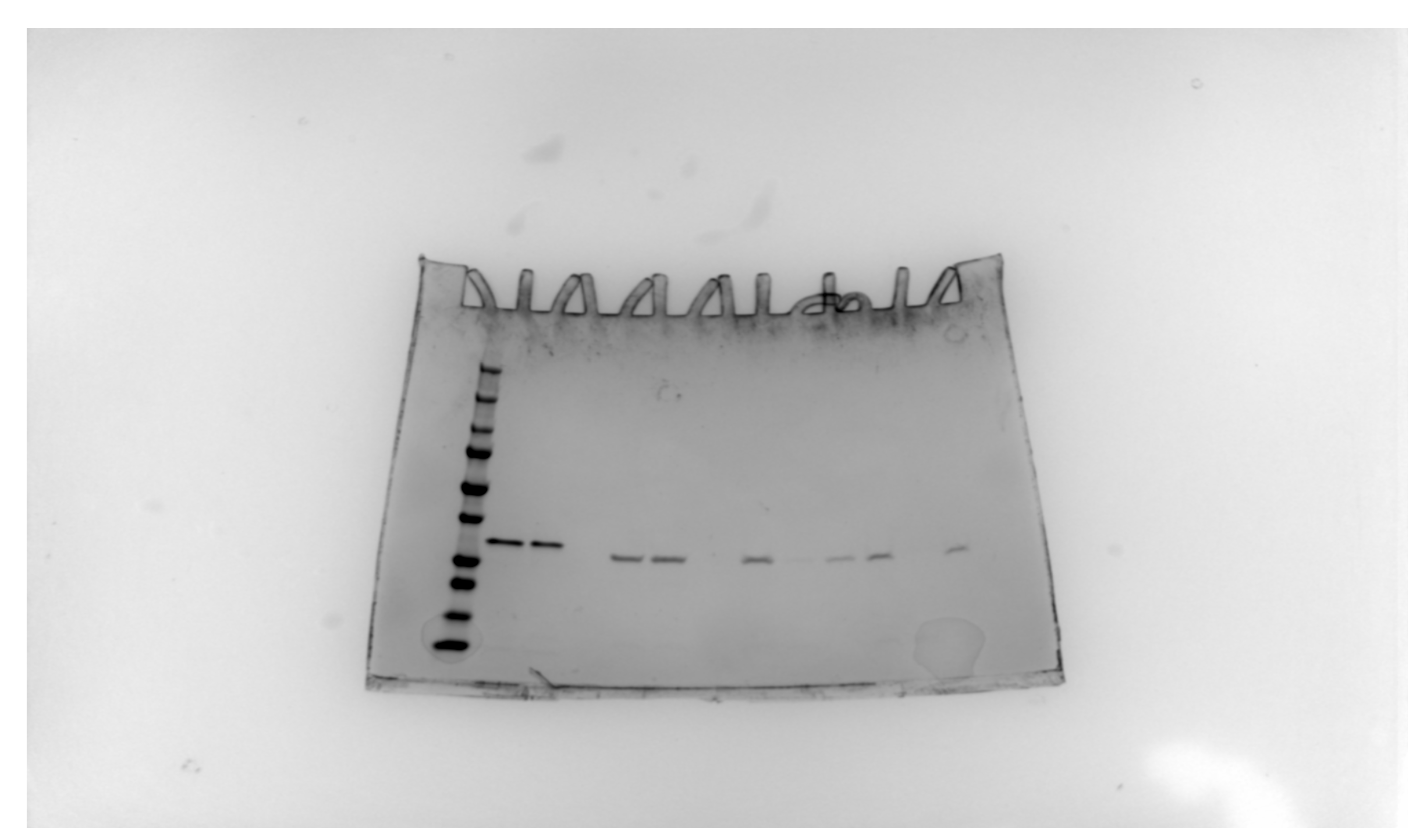

Supplement: Source data 2. [file elife-74206-data2.zip › Raw and annotated gel and blot images 1 of 2/Fig. 2H_raw.png]

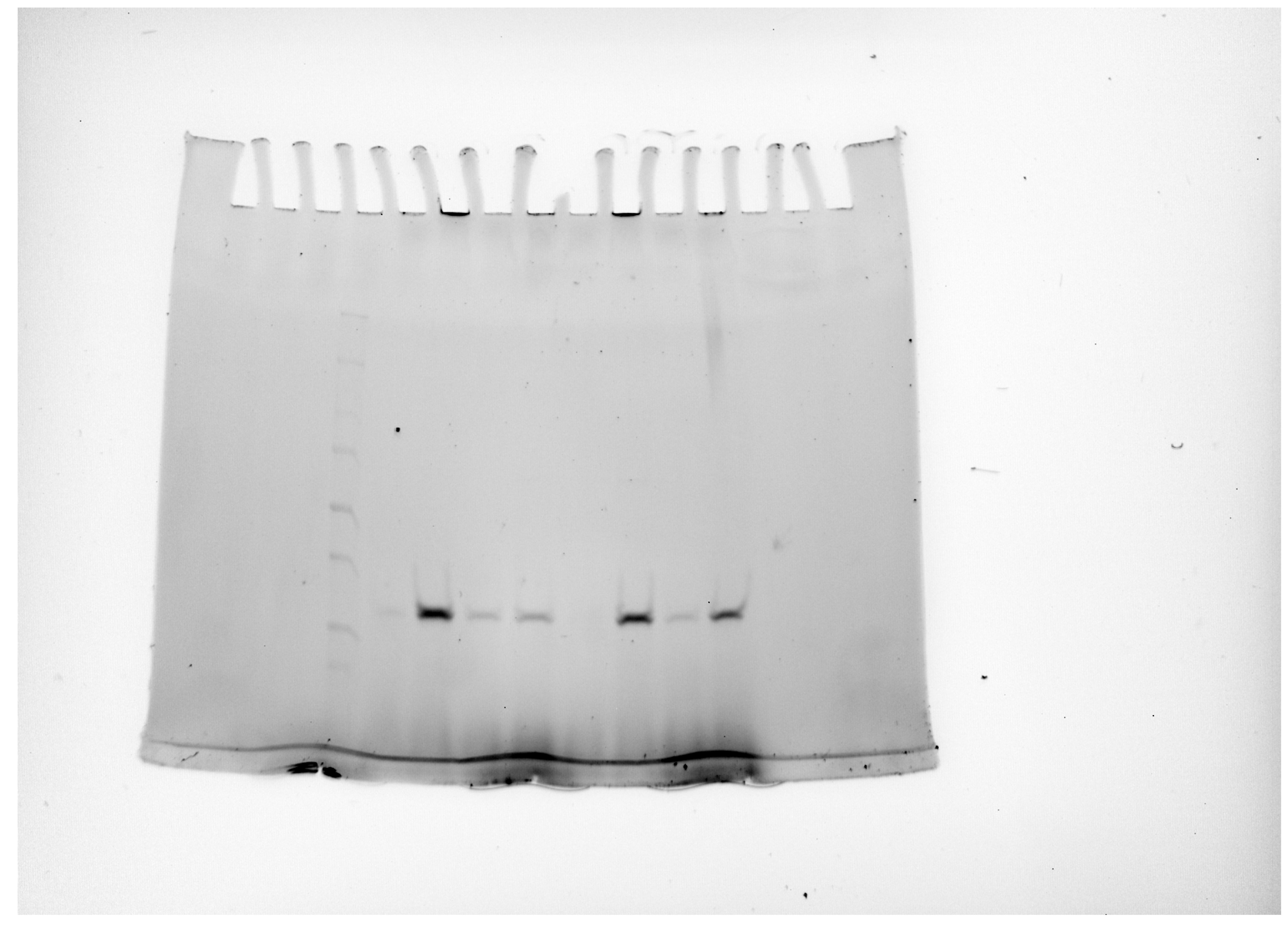

Supplement: Source data 2. [file elife-74206-data2.zip › Raw and annotated gel and blot images 1 of 2/Fig. 2J_raw.png]

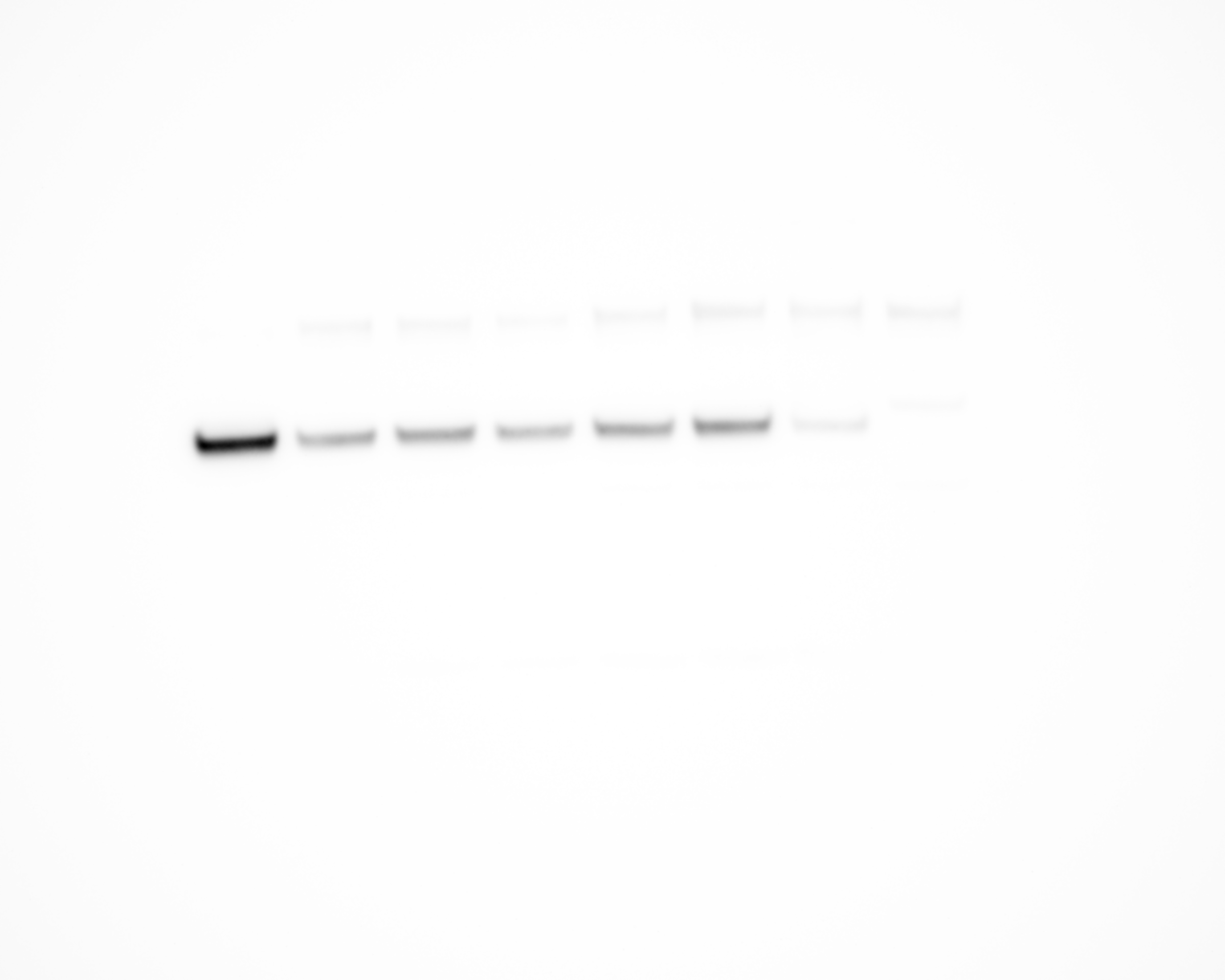

Supplement: Source data 2. [file elife-74206-data2.zip › Raw and annotated gel and blot images 1 of 2/Fig. 1E phospho-p38_raw_Replicate#3.tif]

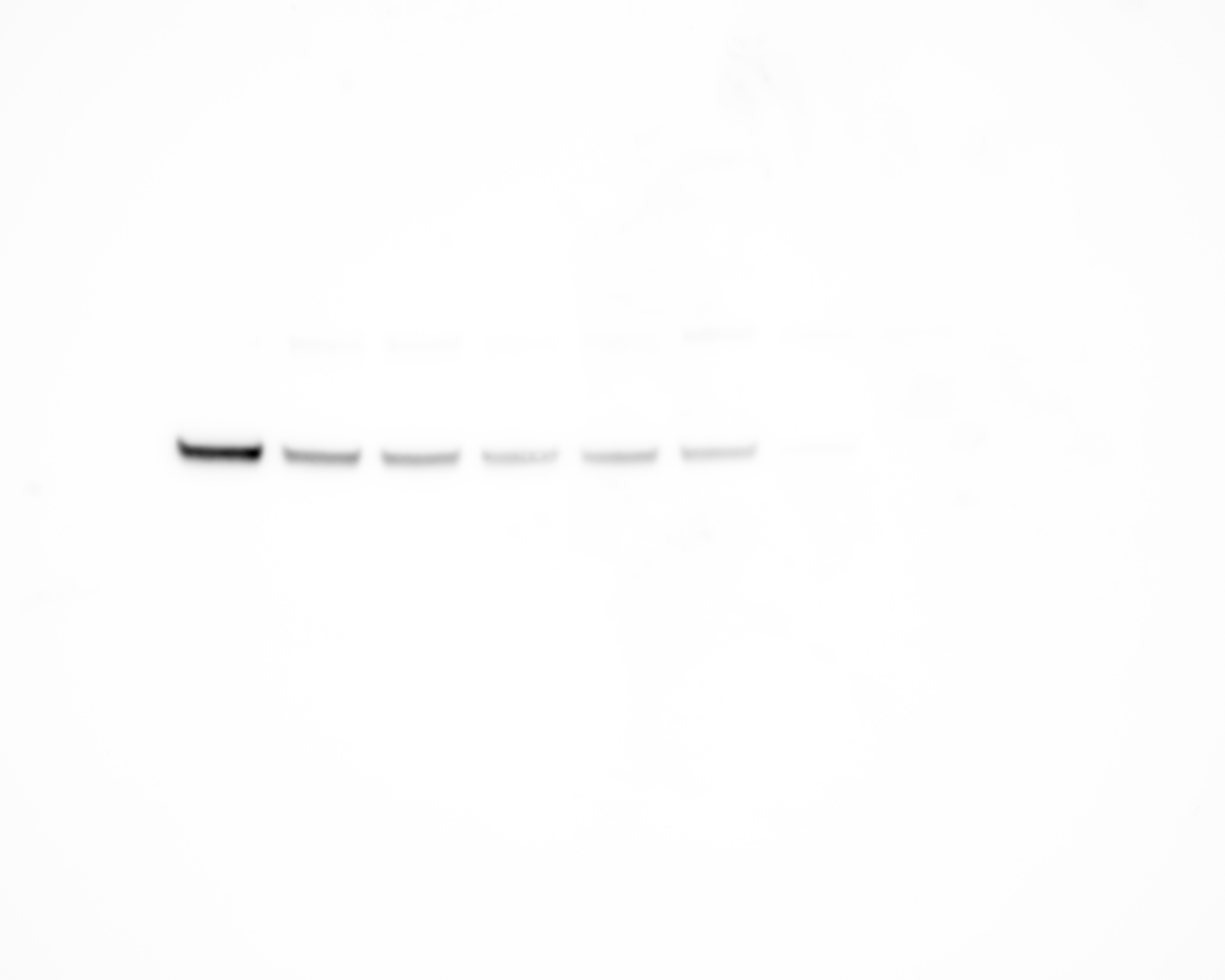

Supplement: Source data 2. [file elife-74206-data2.zip › Raw and annotated gel and blot images 1 of 2/Fig. 1E phospho-p38_raw_Replicate#2.tif]

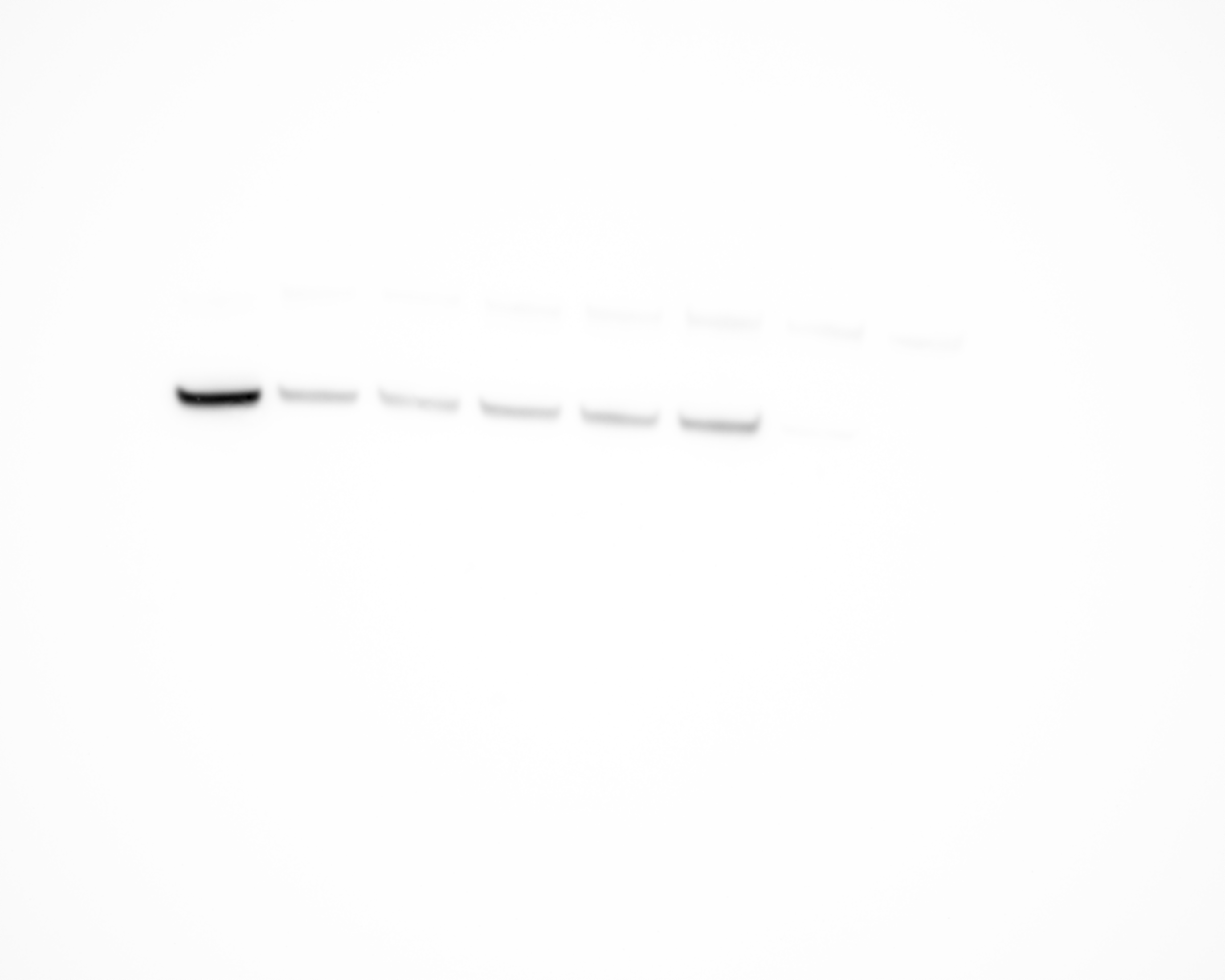

Supplement: Source data 2. [file elife-74206-data2.zip › Raw and annotated gel and blot images 1 of 2/Fig. 1E phospho-p38_raw_Replicate#1.tif]

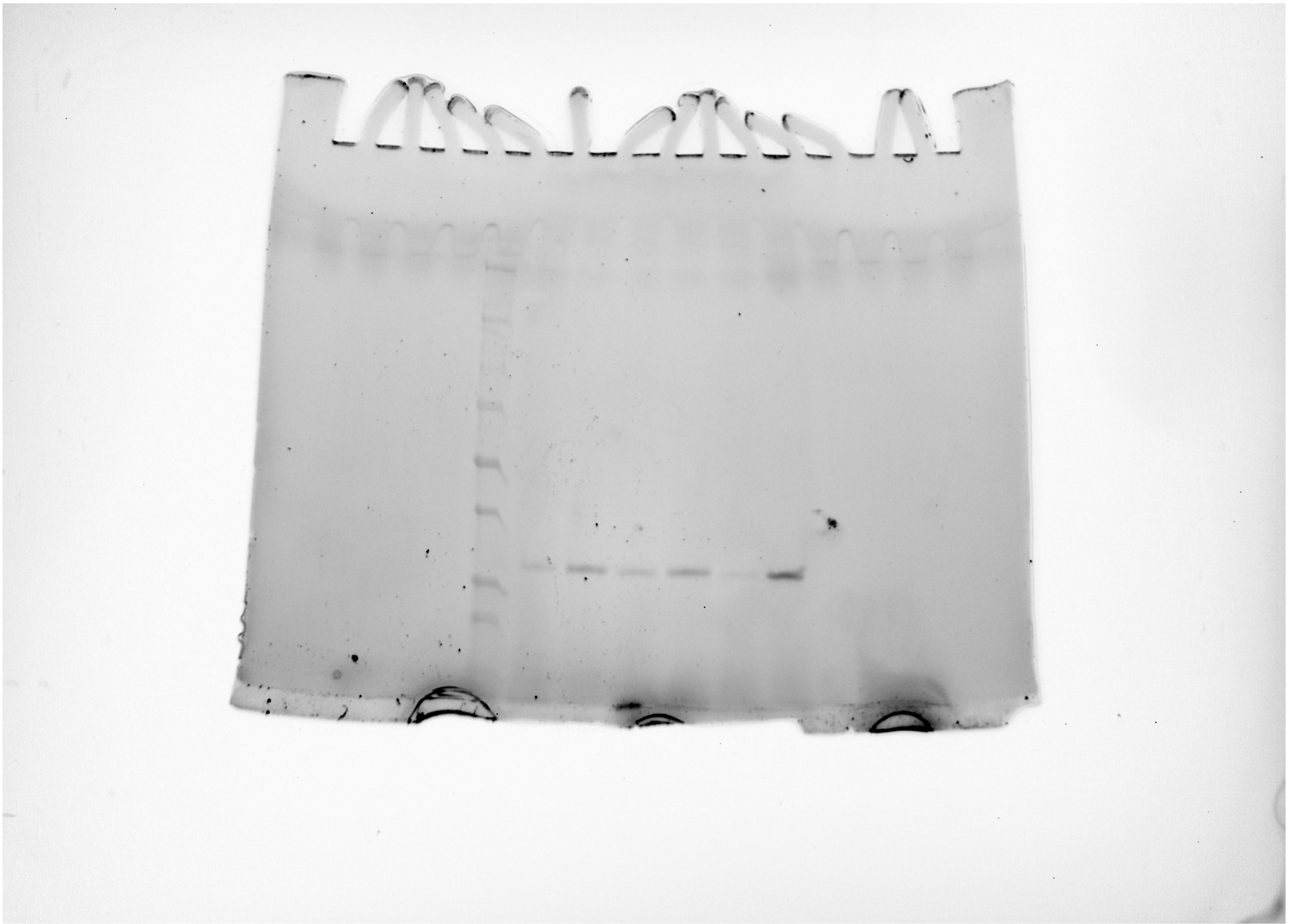

Supplement: Source data 2. [file elife-74206-data2.zip › Raw and annotated gel and blot images 1 of 2/Fig. 2K_raw.png]
