# Supplementary material for: Pathogen infection and cholesterol deficiency activate the C. elegans p38 immune pathway through a TIR-1/SARM1 phase transition
Source: eLife. 2022 Jan 31;11:e74206. doi: 10.7554/eLife.74206 (PMC8923663; doi:10.7554/eLife.74206)
Supplement: Source data 2. [file elife-74206-data2.zip › Raw and annotated gel and blot images 1 of 2/Fig. 2J and 2K_Annotated.pdf]

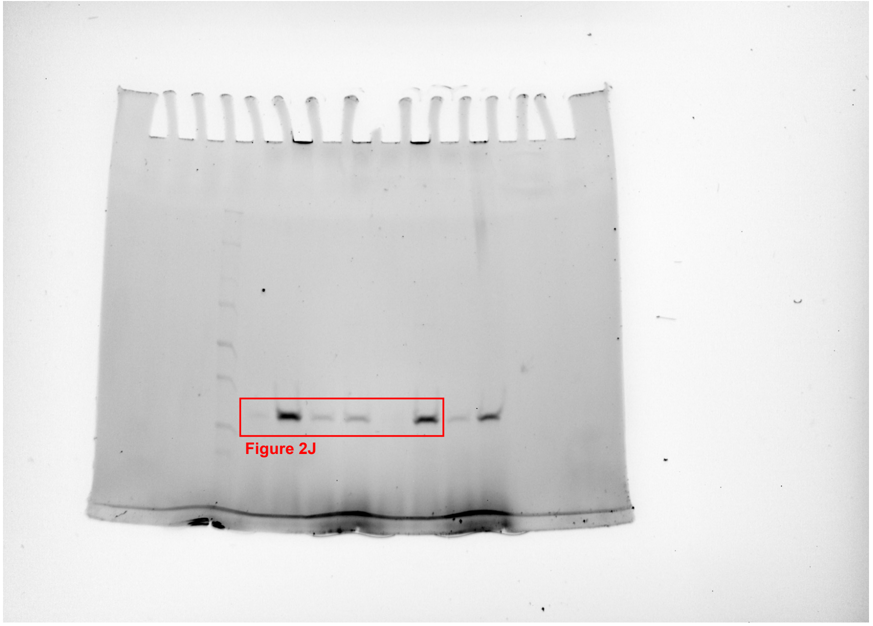

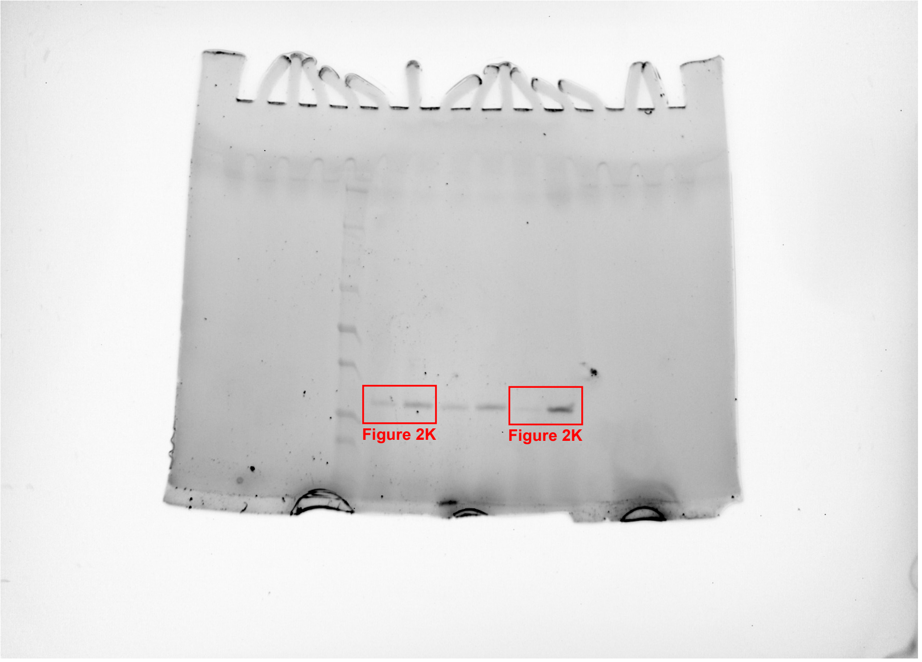

Fig 2J and 2K. (Left) Supernatant and pellet fractions of 10 μM WT and mutant (G747P, E788Q, and H833A) ceTIR in 25% PEG 3350. (Right) Supernatant and pellet fractions of 3 μM WT and mutant (D773N and E788A) ceTIR in 25% PEG 3350. For the images on the right, D773N was not included in the image for the body of the paper as this mutant was not of interest in this paper.
