# Supplementary material for: Pathogen infection and cholesterol deficiency activate the C. elegans p38 immune pathway through a TIR-1/SARM1 phase transition
Source: eLife. 2022 Jan 31;11:e74206. doi: 10.7554/eLife.74206 (PMC8923663; doi:10.7554/eLife.74206)
Supplement: Source data 2. [file elife-74206-data2.zip › Raw and annotated gel and blot images 1 of 2/Fig. 2H_Annotated.pdf]

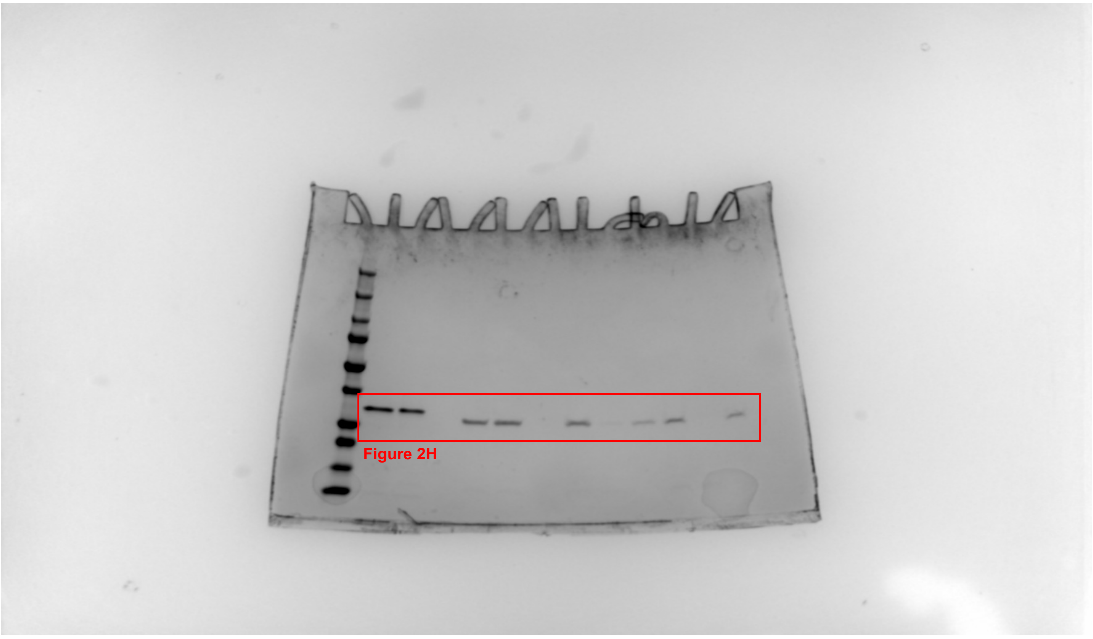

Figure 2H. Precentrifugation control, supernatant, and pelleted fractions of 5 μM ceTIR in 0, 10, 17.5, or 25% PEG 3350.
